# Case Report: Functional validation of a rare variant *BRCA1 c.5193 + 2dupT* in a family with cancer history

**DOI:** 10.3389/fonc.2025.1623700

**Published:** 2025-09-30

**Authors:** Guanlin Dai, Ping Wang, Danqing Wang

**Affiliations:** ^1^ Department of Obstetrics and Gynecology, West China Second University Hospital, Sichuan University, Chengdu, Sichuan, China; ^2^ Key Laboratory of Birth Defects and Related Diseases of Women and Children, Sichuan University, Ministry of Education, Chengdu, Sichuan, China

**Keywords:** *BRCA1*, germline variant, splicing error, hereditary breast and ovarian cancer, variant of uncertain significance

## Abstract

**Background:**

*BRCA1* and *BRCA2* genes are well-established tumor suppressors, crucial for maintaining genomic stability through their roles in DNA repair. Pathogenic variants in *BRCA1/2* genes are implicated in increased susceptibility to breast and ovarian cancers. However, variant interpretation remains challenging due to the large size of *BRCA1/2* (>80 kb) and the broad spectrum of variant forms, particularly for rare or recently identified variants lacking adequate population, functional or segregation data.

**Case presentation:**

This report describes a case of high-grade serous ovarian carcinoma in a patient with a strong family history of cancer. Both her mother and sister died of ovarian cancer. Genetic testing identified the germline variant *BRCA1 c.5193 + 2dupT* both in the patient’s tumor and peripheral blood samples, without other abnormalities detected in genomic homologous recombination deficiency assessment. Her daughter was identified as an unaffected carrier of this variant. Unfortunately, the *BRCA1* status of deceased relatives could not be determined due to the unavailability of samples. Functional studies, including minigene splicing assay and transcript analysis, demonstrated that this variant induces a splicing error, specifically, an aberrant skipping of exon 18, resulting in dysfunction of the *BRCA1*-encoded protein. These findings provide a mechanistic explanation for the observed cancer susceptibility in this family.

**Conclusion:**

This case highlights a rare germline variant, *BRCA1 c.5193 + 2dupT*, in a family with a strong cancer history. *In vitro* functional assays confirmed that this variant induces exon 18 skipping through aberrant splicing, leading to dysfunction of *BRCA1*-encoded protein. To our knowledge, this is the first functional characterization of the variant *BRCA1 c.5193 + 2dupT*, and our data provide novel insights for risk assessment and precision treatment strategies in carriers of this variant.

## 1 Introduction

Breast cancer is the most common malignancy in women worldwide. Ovarian cancer, although less frequent, remains a significant cause of cancer-related death due to late-stage diagnosis. Data from the 2021 global burden of disease, injuries, and risk factors study shows that the global incidence of breast cancer is approximately 2,000,000 cases annually and increases year by year (1). Meanwhile, ovarian cancer accounts for an estimated 200,000 new cases and 100,000 deaths globally each year, ranking the first in mortality among gynecological malignancies (1). *BRCA1* and *BRCA2* genes are critical tumor suppressor genes, playing an important role in homologous recombination mechanism of DNA repair. Variants in these genes can lead to genomic instability, promote tumor cell proliferation and prevent normal cell differentiation, thereby facilitating tumor development (2). Studies have shown that *BRCA1/2* gene variants are relatively common in breast and ovarian cancers (3). Germline variants in *BRCA1/2* gene account for 80% to 90% in cases of hereditary breast and ovarian cancer (HBOC) (3–5). Carriers of pathogenic *BRCA1* variants have a cumulative risk of up to 60% for breast cancer and 59% for ovarian cancer by age 70. Pathogenic *BRCA2* variant carriers, have 55% and 16% risks, respectively. In contrast, the lifetime risks of breast and ovarian cancer in general population are approximately 12% and 1.3%, respectively (5, 6). However, not all *BRCA1/2* variants impair the encoded protein function and variant interpretation remains challenging due to the large size of *BRCA1/2* (>80 kb) the broad spectrum of variant forms.

The American College of Medical Genetics and Genomics/Association for Molecular Pathology (ACMG/AMP) guideline categorizes variants into five classes including pathogenic, likely pathogenic, variant of uncertain significance (VUS), likely benign, and benign based on population data, computational predictions, functional studies, and familial co-segregation data (7, 8). Accurate identification and interpretation of *BRCA1/2* variants are crucial for risk assessment of breast, ovarian and other cancers in women, and serve as important biomarkers for precision treatment. Current sequencing technologies, including Sanger sequencing and next-generation sequencing (NGS), can accurately detect point variants, small insertions, deletions, and rearrangements (9, 10). Advances in sequencing technologies continue to expand the molecular spectrum and drive genomics research. However, the recently identified variants often lack sufficient population and functional data, making their clinical significance unclear and limiting guidance for the clinical management which may result in missed opportunities for early intervention or targeted therapy.

In this report, we detected a highly conserved intronic variant *BRCA1 c.5193 + 2dupT* in a family with a cancer history. We studied the mRNA splicing pattern by constructing a minigene vector *in vitro*, followed by cell transfection and transcript analysis. This is the first report to conduct functional assays *in vitro* to validate the pathogenicity of the variant *BRCA1 c.5193 + 2dupT*, strictly in accordance with the ACMG/AMP variant classification guidelines.

## 2 Case presentation

In November 2023, a 61-year-old female was referred to our department with newly diagnosed ovarian cancer for further systematic treatment, following a recent surgical procedure. She had been diagnosed with high-grade serous ovarian carcinoma (HGSOC) at the local hospital in September 2023 and underwent tumor reduction surgery. Postoperatively, genetic testing was performed using the *BRCA1* and *BRCA2* Gene Mutation Detection Kit, a commercial panel targeting the *BRCA1* and *BRCA2* genes using combinatorial probe-anchor synthesis sequencing technology. Genomic homologous recombination deficiency (HRD) was assessed with the HRD Detection Kit, which qualitatively detects HRD through high-throughput sequencing and a genomic scar analysis algorithm. Library preparation was carried out with reagents supplied in the kits, and sequencing was performed on the DNBSEQ-T7 platform (BGI Biotechnology Co., Ltd., Wuhan, China). The variant *BRCA1 c.5193 + 2dupT* (GRCh37/hg19) was detected in both tumor and peripheral blood samples of this patient. No other pathogenic or likely pathogenic variants were identified.

**Figure 1:**
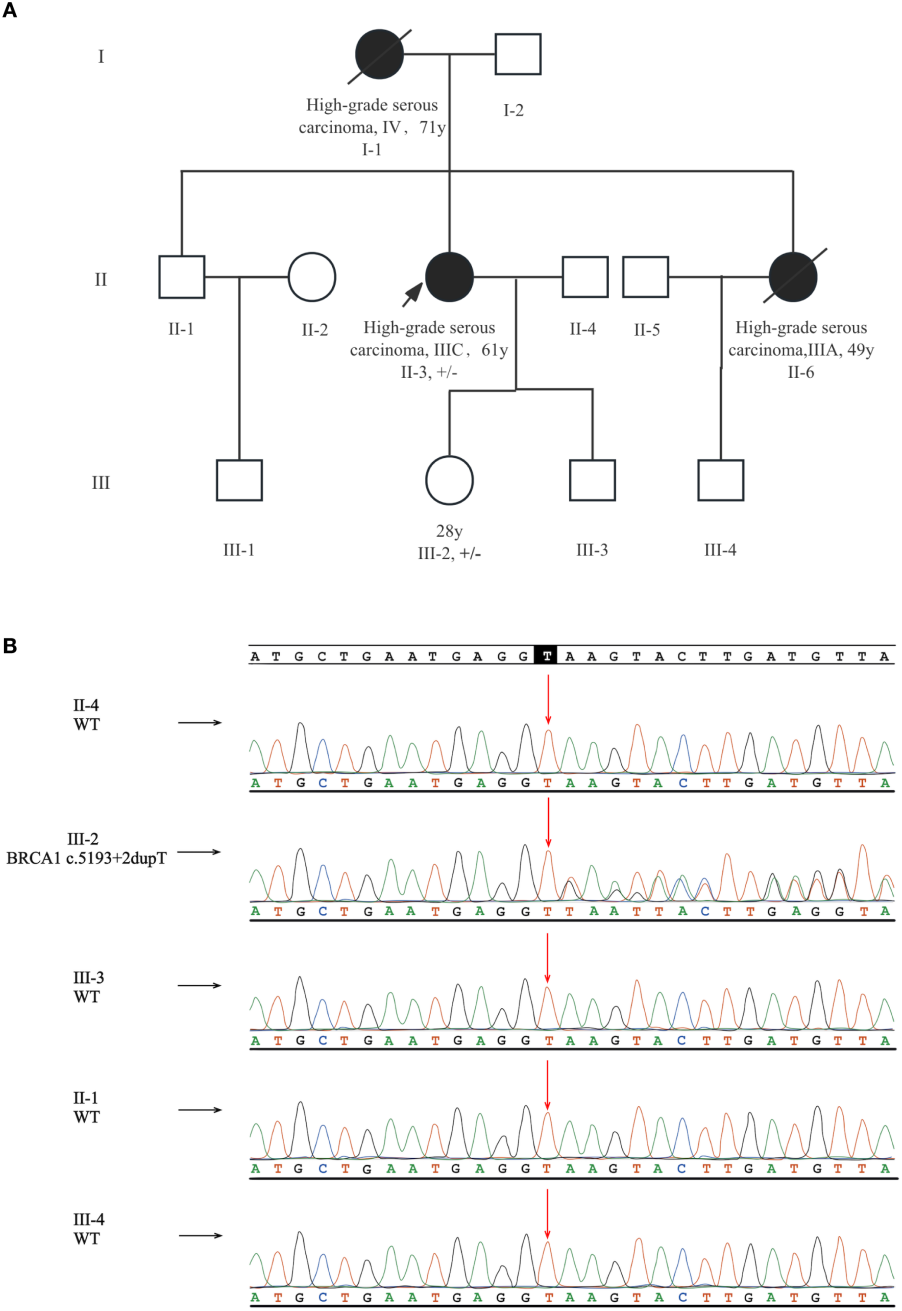
Pedigree and Sanger sequencing results of the family. **(A)** The pedigree of this family with a intronic BRCA1 variant (c.5193 + 2dupT). The proband is marked by an arrow. Patients diagnosed with ovarian cancer are denoted by solid black symbols, and deceased members are marked with a diagonal line. Carriers of the BRCA1 c.5193 + 2dupT variant are annotated in the figure (+/– heterozygous carrier). **(B)** The proband’s daughter was diagnosed as an unaffected carrier.

This patient reported a typical family history of ovarian cancer. Her mother (I-1) was diagnosed with HGSOC at the age of 71 and unfortunately passed away due to this cancer at age 75. Her sister (II-6) was also diagnosed with HGSOC at age 49 and succumbed to ovarian cancer three years post-surgery. The pedigree chart is detailed in **Figure 1A**. As of November 2023, no other family members had reported a history of cancer. To further evaluate this variant in family members, Sanger sequencing was performed, and the patient’s daughter (III-2) was identified as an unaffected heterozygous carrier. While it was absent in other relatives (**Figure 1B**). Her mother (I-1) and sister (II-6) had died, and thus failed to perform sequencing.

The variant *BRCA1 c.5193 + 2dupT*, located in intron 19, is well conserved and has not been recorded in population databases such as the Exome Aggregation Consortium, 1000 Genomes Project, and the Exome Variant Server. In the ClinVar database and previous literature, this variant was classified discordantly as pathogenic or of uncertain significance, without any functional assessment and familial co-segregation analysis. Based on existing data at that time, the pathogenicity of *BRCA1 c.5193 + 2dupT* was unclear and could only be classified as a VUS according to the ACMG/AMP guideline.

**Figure 2:**
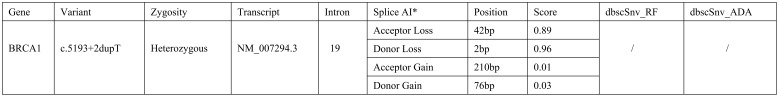
Bioinformatic Predictions of Splicing Disruption for BRCA1 c.5193 + 2dupT Variant. *The figure shows the SpliceAI output, including delta scores for donor and acceptor site loss. The delta scores indicate the probability of splicing disruption at the donor and acceptor sites.

Traditional in-silicon prediction algorithms, including dbscSNV_ADA and dbscSNV_RF, were applied but yielded no positive results. However, SpliceAI, a deep-learning model, suggested that variant *BRCA1 c.5193 + 2dupT* could disrupt splicing process, causing a 2 bp loss at the splicing donor site with a score of 0.96, and a 42 bp loss at the acceptor site with a score of 0.89 (**Figure 2**). This could result in the aberrant transcript and the consequent loss of encoded protein function. Nevertheless, these predictions were solely based on machine learning, and are insufficient to support its pathogenicity.

**Figure 3:**
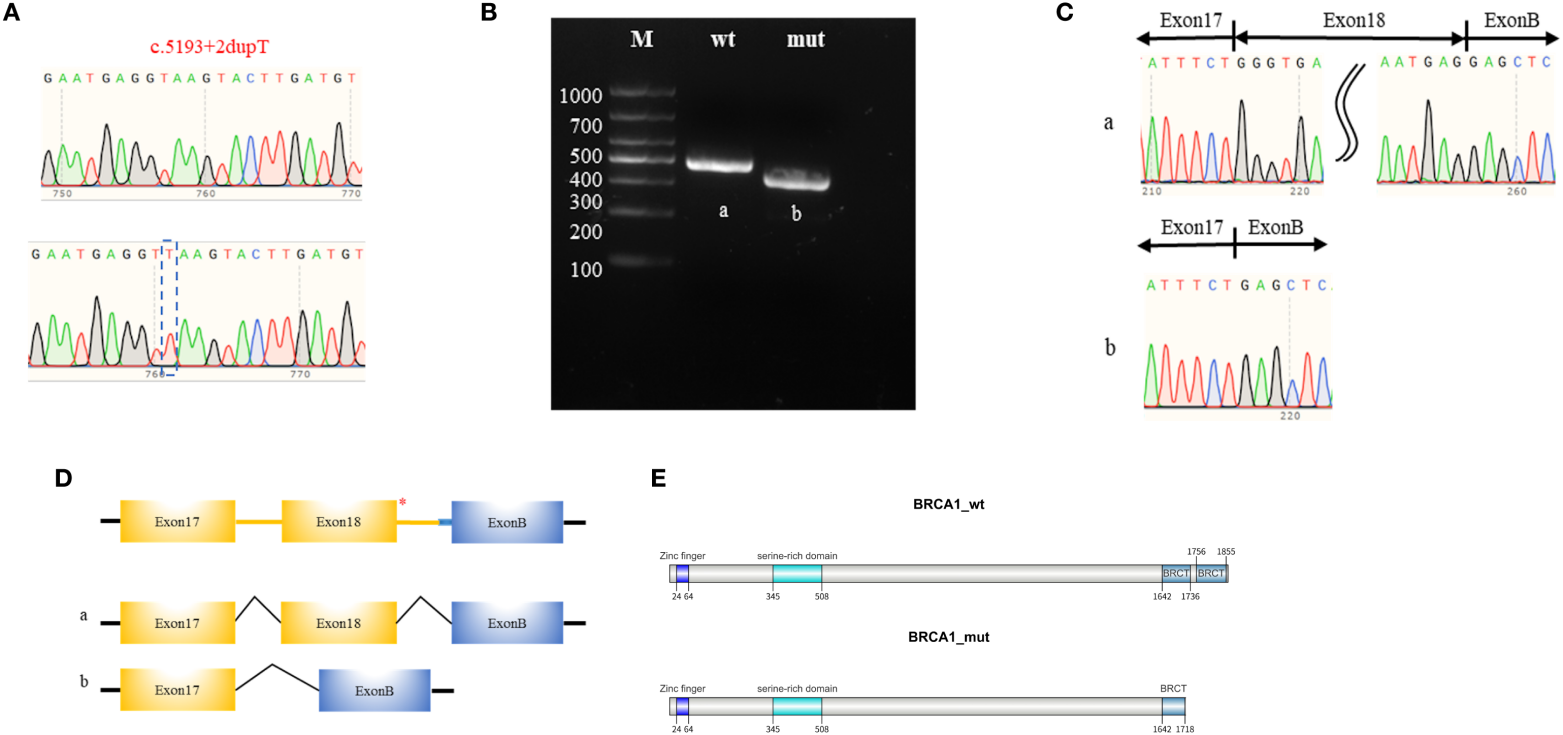
Minigene splicing assay and transcripts analysis. **(A)** Sanger sequencing chromatogram of the pcMINI-C-BRCA1-wt/mut plasmids. **(B)** Agarose gel electrophoresis of the transcription products, with the respective bands labeled as ‘a’ for the wild type and ‘b’ for the mutant. **(C)** Sanger sequencing chromatogram of the transcription bands. **(D)** Schematic diagram of the pcMINI-C-BRCA1 plasmids which contains a universal Exon B (57 base pairs, 57 bp). The asterisk (*) indicates the variant site. The schematic diagrams of the RNA splicing patterns for the wild-type (a) and the mutant-type (b). **(E)** The transcript NM_007294.3 with exon 18 skipping acquires a premature termination codon which results in the production of a truncated BRCA1 protein (1718 amino acids) instead of the wild-type protein (1863 amino acids), thereby impairing protein function due to the loss of the C-terminal region.

To further validate these predictions, we performed a minigene splicing assay and transcript analysis. Human genomic DNA fragment including the splicing sites of exon 17 and 18 (*BRCA1* RNA F: 5’-TGAATGAGGTTAAGTACTTGA; *BRCA1* RNA R: 5’-TCAAGTACTTAACCTCATTCA) served as the template and was cloned into pcMINI-C vector for plasmid reconstruction (**Supplementary Figure 1**). Sequencing diagrams of the constructed plasmids were depicted in **Figure 3A**. The reconstructed plasmids were transfected into human 293T cells, and RNA samples were extracted 24 hours later for RT-PCR analysis. The aberrant transcript product in the *BRCA1 c.5193 + 2dupT* carrier was clearly identified with a distinct band by agarose gel electrophoresis. (**Figure 3B**). These products were further validated by Sanger sequencing (**Figure 3C**). These results indicated that the *BRCA1 c.5193 + 2dupT* variant can disrupt the splicing pattern, leading to the skipping of exon 18. The specific splicing patterns are shown in **Figure 3D**. We further organized the coding sequences of *BRCA1* wild-type and variant-type of exon 18 skipping, as well as the corresponding amino acid sequences. Details have been provided in the Supplementary Material. Due to exon 18 skipping, transcript NM_007294.3 acquires a premature termination codon. This results in a truncated *BRCA1*-encoded protein (1718 amino acids) instead of the wild-type (1863 amino acids), thereby impairing protein function due to the loss of the C-terminal region. The schematic diagram is shown in **Figure 3E**.

**Figure 4:**
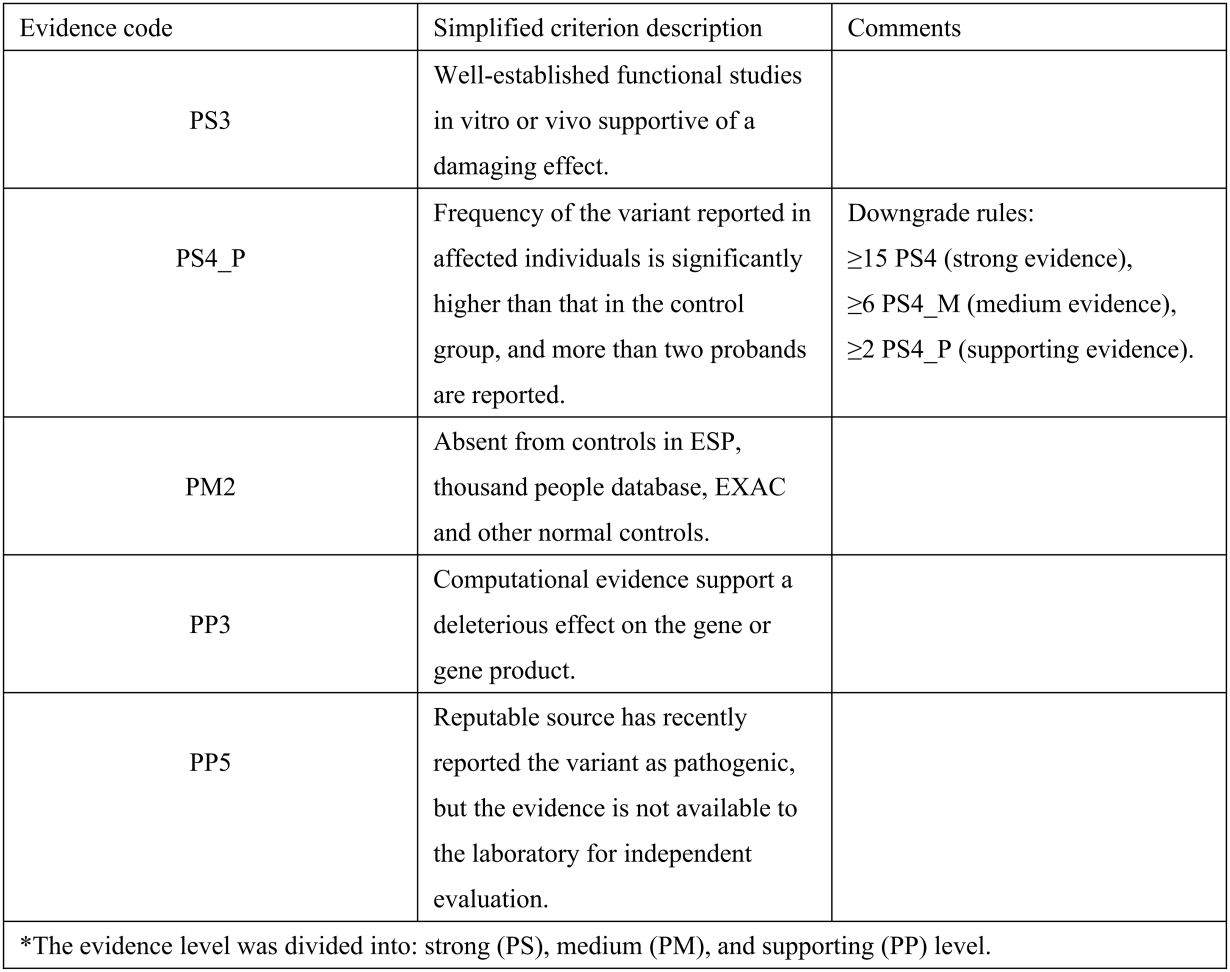
Evidence for the classification of BRCA1 c.5193 + 2dupT as likely pathogenic based on the ACMG/AMP variant classification guidelines.

Based on these findings, we reclassified this variant strictly in accordance with the ACMG/AMP guidelines. This variant meets criteria PS3 (splicing error confirmed by functional assay), PM2 (absent in normal controls), PS4_P (reported in more than 2 probands), PP3 (computational support) and PP5 (previously reported in ClinVar), warranting reclassification into “likely pathogenic”. The specific basis for reclassification is summarized in **Figure 4**. These results provide a mechanistic explanation for the cancer susceptibility within this family.

## 3 Discussion

In this report, we proposed and confirmed for the first time that *BRCA1 c.5193 + 2dupT* variant disrupts splicing pattern, causing the skipping of exon 18 in transcripts. Due to the exon 18 skipping, the transcript (NM_007294.3) acquires a premature termination codon. This results in a truncated *BRCA1-encoded* protein, thereby impairing protein function due to the loss of the C-terminal region. This report integrates the results from familial co-segregation analysis, computational prediction, and functional assays *in vitro*, providing a mechanistic explanation for the cancer susceptibility within this family.

RNA splicing is an essential biological process in eukaryotic gene expression, precisely removing introns from precursor mRNA through the recognition of cis-acting elements (11). Accurate RNA splicing relies on both canonical splice signals (CSSs) including splice donor and acceptor sites, and auxiliary splicing regulatory elements (SREs), including exonic or intronic splicing enhancers and silencers (12). Single-nucleotide variants can disrupt these signals or elements, leading to aberrant splicing events, manifesting as exon skipping, intron retention or activation of cryptic splice sites (13). These events often generate transcripts with premature termination codons, frameshift variants, or in-frame deletions/insertions, which may result in structural or functional abnormalities of the encoded proteins (13). Abnormal splicing events have been implicated in the pathogenesis of genetic disorders and cancers, accounting for up to 60% in inherited monogenic disorders (14). Although 70%-80% of pathogenic splice events are caused through CSSs disruptions, most variants outside these regions such as those affecting SREs, remain undiagnosed (15). Previously reported the *BRCA1 c.5080>T* variant leads to the skipping of exon 18 through disrupting a splicing enhancer (16). Our findings extend this paradigm, demonstrating that a single duplicated nucleotide within an intronic region can also interfere with the splicing process, resulting in the aberrant skipping of exon 18. These findings underscore the necessity of evaluating both canonical and non-canonical splicing regions in *BRCA1* gene.

Functional analysis is crucial for interpreting the biological significance of variants, especially those in non-canonical splicing regions. In cases of HBOC, molecular profiling analysis, particularly of the *BRCA1* and *BRCA2* genes, assists in risk assessment and targeted therapies. However, most diagnosed variants are identified solely through DNA sequencing. Variants in non-canonical splicing regions, due to the lack of sufficient population and functional data, are often classified as VUS, which cannot provide guidance for the clinical management (17). Thus, the 9% of splicing variations previously reported in the Human Gene Mutation Database were undoubtedly underestimated (18). For patients with a potential family history of cancer, further functional analysis of variants with unknown significance is essential.

Computational prediction serves as a preliminary screening tools for functional analysis. Traditional tool, such as dbscSNV_ADA and dbscSNV_RF, are mainly based on traditional machine learning algorithms like adaptive boosting and random forest, relying on existing data of splicing sites and splicing signal. Their prediction performs well on variants at CSSs, including splicing donor (+1 and +2) and acceptor (-1 and -2) sites (19). If the variants in question fall outside the designed scope, it could yield negative results. SpliceAI, a 32-layer deep neural network trained on more diverse and extensive datasets that may include examples similar to the variant being studied, can accurately predict the impact of variants on splicing sites, especially for non-canonical or deep intronic variants (20). In contrast, traditional tools, lacking such extensive training datasets, may fail to detect splicing events in these cases. The output of SpliceAI typically includes the results with delta scores which indicate the probability of function disruption of variant sites (ranging from 0 to 1). A delta score above 0.8 typically signifies a high likelihood of splicing disruption (21). In this report, SpliceAI predicted that the *BRCA1 c.5193 + 2dupT* variant would cause a 2 bp loss at the donor site with a score of 0.96 and a 42 bp loss at the acceptor site with a score of 0.89. These scores suggest that the variant may severely impair splice site function, result in detrimental impact on the transcript product and encoded protein, meeting the PP3 criteria in ACMG/AMP guidelines.

Although spliceAI has outperformed in this report, positive results from computational prediction alone remain insufficient for the pathogenic classification of a variant. Functional assays, such as minigene splicing assays, can directly observe the impact of variants on RNA splicing and provide authentic biological evidence. The minigene splicing assay, analyzing the splicing outcome of a single allele, is a powerful tool for evaluating allele-specific expression. It can demonstrate that the variant allele produces abnormal transcripts that are predicted to disrupt the encoded protein’s structure and function. This is a crucial step in classifying splicing variants as pathogenic and can be exemplified by the variant *BRCA1 c.5193 + 2dupT* in this report, where computational tools and clinical databases exhibited discordant interpretations. Our minigene splicing assay and RT-PCR analysis directly demonstrated the aberrant splicing events, resolving this ambiguity through functional evidence. Similarly, the variant *BRCA1 c.5152 + 5G>C* was initially classified as VUS until a minigene assay confirmed the aberrant skipping of exon 17 (22). The variant *BRCA1 c.442–7 T>A*, with conflicting results in multiple computational predictions, was further confirmed by minigene splicing assay to cause a 5-nt insertion before exon 8: TTTAG in the transcript (23). The variant *BRCA1 c.231 G>T* was predicted to have no effect in computational prediction, while minigene assay revealed the exon 6 skipping in transcripts (23). The variant *c.5193 + 2T>C*, located at CSS and multiple computational algorithms have predicted that this variant might disrupt the donor site. While minigene splicing assay revealed no differences in the transcription products between this variant and the wild-type allele (23). In these cases, employing additional methods to ascertain the functional impact of the variants appears to be essential. RNA sequencing can directly analyze transcripts through high-throughput sequencing and detect aberrant splicing events (24). Due to the availability and stability of RNA in tumor or blood samples, RNA sequencing is rarely included in the routine molecular diagnostics. Moreover, when multiple suspected variants are present in a single allele, minigene splicing assays are still needed to elucidate the causal relationship between variants and aberrant transcripts. In this report, the unavailability of RNA data from the proband limited our direct assessment of the variant’s impact on transcripts. Nevertheless, we conducted a minigene splicing assay to simulate the transcription process *in vitro* and analyzed the transcript products, thereby indirectly confirming the aberrant splicing event caused by this variant. Current ACMG/AMP guidelines prioritize functional evidence for splicing variant classification, and minigene splicing assays serve as a critical part in fulfilling these evidence requirements (8).

In this report, the unavailability of biological samples from the deceased relatives precluded us from providing rigorous segregation data, and fulfilling the stringent PS4 criteria in the ACMG/AMP guideline. The latest ACMG/AMP guideline suggests that for extremely rare variants, case-control studies may not be statistically significant and if a variant originally observed in multiple patients with the same phenotype (and absent in controls) would have qualified a downgraded PS4 criteria from the strong evidence of pathogenicity to a moderate or supporting level of evidence (36). The downgrade rules are as follows: ≥15 probands for PS4 (strong evidence), ≥6 probands for PS4_M (moderate evidence), and ≥2 probands for PS4_P (supporting evidence). Based on sporadic cases recorded in the database and confirmed cases reported in this report, the frequency of the variant *BRCA1 c.5193 + 2dupT* in the affected population is significantly higher than that in the control population, meeting the ACMG/AMP criteria for evidence downgrading. Therefore, PS4 can be downgraded to the supporting level of evidence (PS4_P). The pathogenicity classification evidence for this variant ultimately includes one strong (PS3), one moderate (PM2), and three supporting (PS4_P, PP3, and PP5) pieces of evidence, still qualifying it as a likely pathogenic variant according to the ACMG/AMP guideline. While the lack of genetic data weakens the segregation evidence in this report, PS4_P still provides meaningful support for its pathogenicity classification.

The *BRCA1* protein, crucial for in maintaining genomic stability, primarily exerts its tumor-suppressive function by mediating the homologous recombination repair mechanism for DNA double-strand breaks (2). Splicing variants in BRCA1 can lead to homologous recombination deficiency, significantly increasing genomic instability and driving tumorigenesis (2). Germline variants in *BRCA1* gene, classified as pathogenic or likely pathogenic, confer markedly elevated lifetime cancer risks, with breast cancer risk reaching 60% and ovarian cancer risk reaching 59% (5, 6). Therefore, *BRCA1* gene variants can be used for risk assessment of breast, ovarian and other cancers. Given that *BRCA1/2* germline variants are inherited in an autosomal dominant manner, genetic counselling and testing are recommended for the first-degree relatives of the proband with risk management based on the screening results (9, 25). Unaffected carriers of pathogenic or likely pathogenic variants in *BRCA1* should undergo a standardized surveillance procedure in accordance with their age and reproductive options. Females should initiate the consistent breast self-examination starting at age 18 and undergo clinical breast examination every 6–12 months beginning at age 25 (26). From age 25 onward, regular breast imaging surveillance should be implemented, with breast MRI preferred due to the established radiation-associated risk in carriers (27). Furthermore, risk-reducing mastectomy and chemopreventive agents require individual risk-benefit assessment to thoroughly weigh the interventions’ advantages against potential risk (26). The risk management of ovarian cancer requires a comprehensive consideration integrating cancer risk reduction, fertility preservation, and management of hormone-related symptoms. Current primary screening tools include CA125 and pelvic ultrasound, and definitive surgical risk reduction via bilateral salpingo-oophorectomy is recommended between ages 35 and 40 years following completion of childbearing (26, 28).

*BRCA1* gene variants are also critical biomarkers for precision treatment. Studies have shown that ovarian cancer patients with pathogenic *BRCA* variants are more sensitive to platinum-based chemotherapy and can benefit from treatment with poly (ADP-ribose) polymerase (PARP) inhibitors (29). The efficacy and safety of PARP inhibitors including Olaparib, niraparib, rucaparib, and talazoparib, have been demonstrated in patients with breast or ovarian cancer carrying pathogenic *BRCA1/2* variants (29–32). Therefore, conducting *BRCA* variant testing and functional interpretation for patients with breast or ovarian cancer is beneficial for devising precision treatment plans. In addition to *BRCA1/2* genes, other homologous recombination repair genes are also implicated in breast and ovarian cancers, such as *PALB2*, *ATM*, and *CHEK2* (33–35). Variants in these genes may also increase the risk of breast and ovarian cancers. Therefore, genetic testing and counselling is extremely important in clinical practice. Early detection and management of breast and ovarian cancer risks can help improve patients’ prognosis and quality of life.

Accurate interpretation and reclassification of the variant *BRCA1 c.5193 + 2dup*T has significant clinical implications for this family. It provides a mechanistic explanation for their observed cancer susceptibility and enables more informed decisions regarding surveillance, risk-reducing interventions, and targeted therapies. This case underscores the importance of integrating functional studies with genetic counselling and highlights the need for further research to assess the clinical significance of VUS in *BRCA1/2* genes. This study also provides a novel research approach under the condition that clinical samples are still lacking for the discovery of rare *BRCA1/2* variants in the clinic. Future research should focus on developing comprehensive databases and functional assays to address the challenges posed by VUS, ultimately improving clinical outcomes for patients and families affected by hereditary cancer syndromes.

## 4 Conclusion

In conclusion, this study provides functional evidence for the likely pathogenicity of the variant *BRCA1 c.5193 + 2dupT*, emphasizing the importance of integrating computational predictions with functional validation in variant interpretation. These findings have significant clinical implications for the carriers, enabling more informed risk assessment and management strategies. Future research should focus on developing comprehensive databases and functional assays to address the challenges posed by VUSs, ultimately improving clinical outcomes for patients and families affected by hereditary cancer syndromes.

## Data Availability

The raw data supporting the conclusions of this article will be made available by the authors, without undue reservation.
