# Supplementary material for: Case Report: Functional validation of a rare variant BRCA1 c.5193 + 2dupT in a family with cancer history
Source: Front Oncol. 2025 Sep 30;15:1623700. doi: 10.3389/fonc.2025.1623700 (PMC12518100; doi:10.3389/fonc.2025.1623700)
Supplement: Supplementary file 1 [file DataSheet1.zip › Supplementary Material/BRCA1-the coding sequence and amino acid sequences.docx]

BRCA1-wild type coding sequence

ATGGATTTATCTGCTCTTCGCGTTGAAGAAGTACAAAATGTCATTAATGCTATGCAGAAAATCTTAGAGTGTCCCATCTGTCTGGAGTTGATCAAGGAACCTGTCTCCACAAAGTGTGACCACATATTTTGCAAATTTTGCATGCTGAAACTTCTCAACCAGAAGAAAGGGCCTTCACAGTGTCCTTTATGTAAGAATGATATAACCAAAAGGAGCCTACAAGAAAGTACGAGATTTAGTCAACTTGTTGAAGAGCTATTGAAAATCATTTGTGCTTTTCAGCTTGACACAGGTTTGGAGTATGCAAACAGCTATAATTTTGCAAAAAAGGAAAATAACTCTCCTGAACATCTAAAAGATGAAGTTTCTATCATCCAAAGTATGGGCTACAGAAACCGTGCCAAAAGACTTCTACAGAGTGAACCCGAAAATCCTTCCTTGCAGGAAACCAGTCTCAGTGTCCAACTCTCTAACCTTGGAACTGTGAGAACTCTGAGGACAAAGCAGCGGATACAACCTCAAAAGACGTCTGTCTACATTGAATTGGGATCTGATTCTTCTGAAGATACCGTTAATAAGGCAACTTATTGCAGTGTGGGAGATCAAGAATTGTTACAAATCACCCCTCAAGGAACCAGGGATGAAATCAGTTTGGATTCTGCAAAAAAGGCTGCTTGTGAATTTTCTGAGACGGATGTAACAAATACTGAACATCATCAACCCAGTAATAATGATTTGAACACCACTGAGAAGCGTGCAGCTGAGAGGCATCCAGAAAAGTATCAGGGTAGTTCTGTTTCAAACTTGCATGTGGAGCCATGTGGCACAAATACTCATGCCAGCTCATTACAGCATGAGAACAGCAGTTTATTACTCACTAAAGACAGAATGAATGTAGAAAAGGCTGAATTCTGTAATAAAAGCAAACAGCCTGGCTTAGCAAGGAGCCAACATAACAGATGGGCTGGAAGTAAGGAAACATGTAATGATAGGCGGACTCCCAGCACAGAAAAAAAGGTAGATCTGAATGCTGATCCCCTGTGTGAGAGAAAAGAATGGAATAAGCAGAAACTGCCATGCTCAGAGAATCCTAGAGATACTGAAGATGTTCCTTGGATAACACTAAATAGCAGCATTCAGAAAGTTAATGAGTGGTTTTCCAGAAGTGATGAACTGTTAGGTTCTGATGACTCACATGATGGGGAGTCTGAATCAAATGCCAAAGTAGCTGATGTATTGGACGTTCTAAATGAGGTAGATGAATATTCTGGTTCTTCAGAGAAAATAGACTTACTGGCCAGTGATCCTCATGAGGCTTTAATATGTAAAAGTGAAAGAGTTCACTCCAAATCAGTAGAGAGTAATATTGAAGACAAAATATTTGGGAAAACCTATCGGAAGAAGGCAAGCCTCCCCAACTTAAGCCATGTAACTGAAAATCTAATTATAGGAGCATTTGTTACTGAGCCACAGATAATACAAGAGCGTCCCCTCACAAATAAATTAAAGCGTAAAAGGAGACCTACATCAGGCCTTCATCCTGAGGATTTTATCAAGAAAGCAGATTTGGCAGTTCAAAAGACTCCTGAAATGATAAATCAGGGAACTAACCAAACGGAGCAGAATGGTCAAGTGATGAATATTACTAATAGTGGTCATGAGAATAAAACAAAAGGTGATTCTATTCAGAATGAGAAAAATCCTAACCCAATAGAATCACTCGAAAAAGAATCTGCTTTCAAAACGAAAGCTGAACCTATAAGCAGCAGTATAAGCAATATGGAACTCGAATTAAATATCCACAATTCAAAAGCACCTAAAAAGAATAGGCTGAGGAGGAAGTCTTCTACCAGGCATATTCATGCGCTTGAACTAGTAGTCAGTAGAAATCTAAGCCCACCTAATTGTACTGAATTGCAAATTGATAGTTGTTCTAGCAGTGAAGAGATAAAGAAAAAAAAGTACAACCAAATGCCAGTCAGGCACAGCAGAAACCTACAACTCATGGAAGGTAAAGAACCTGCAACTGGAGCCAAGAAGAGTAACAAGCCAAATGAACAGACAAGTAAAAGACATGACAGCGATACTTTCCCAGAGCTGAAGTTAACAAATGCACCTGGTTCTTTTACTAAGTGTTCAAATACCAGTGAACTTAAAGAATTTGTCAATCCTAGCCTTCCAAGAGAAGAAAAAGAAGAGAAACTAGAAACAGTTAAAGTGTCTAATAATGCTGAAGACCCCAAAGATCTCATGTTAAGTGGAGAAAGGGTTTTGCAAACTGAAAGATCTGTAGAGAGTAGCAGTATTTCATTGGTACCTGGTACTGATTATGGCACTCAGGAAAGTATCTCGTTACTGGAAGTTAGCACTCTAGGGAAGGCAAAAACAGAACCAAATAAATGTGTGAGTCAGTGTGCAGCATTTGAAAACCCCAAGGGACTAATTCATGGTTGTTCCAAAGATAATAGAAATGACACAGAAGGCTTTAAGTATCCATTGGGACATGAAGTTAACCACAGTCGGGAAACAAGCATAGAAATGGAAGAAAGTGAACTTGATGCTCAGTATTTGCAGAATACATTCAAGGTTTCAAAGCGCCAGTCATTTGCTCCGTTTTCAAATCCAGGAAATGCAGAAGAGGAATGTGCAACATTCTCTGCCCACTCTGGGTCCTTAAAGAAACAAAGTCCAAAAGTCACTTTTGAATGTGAACAAAAGGAAGAAAATCAAGGAAAGAATGAGTCTAATATCAAGCCTGTACAGACAGTTAATATCACTGCAGGCTTTCCTGTGGTTGGTCAGAAAGATAAGCCAGTTGATAATGCCAAATGTAGTATCAAAGGAGGCTCTAGGTTTTGTCTATCATCTCAGTTCAGAGGCAACGAAACTGGACTCATTACTCCAAATAAACATGGACTTTTACAAAACCCATATCGTATACCACCACTTTTTCCCATCAAGTCATTTGTTAAAACTAAATGTAAGAAAAATCTGCTAGAGGAAAACTTTGAGGAACATTCAATGTCACCTGAAAGAGAAATGGGAAATGAGAACATTCCAAGTACAGTGAGCACAATTAGCCGTAATAACATTAGAGAAAATGTTTTTAAAGAAGCCAGCTCAAGCAATATTAATGAAGTAGGTTCCAGTACTAATGAAGTGGGCTCCAGTATTAATGAAATAGGTTCCAGTGATGAAAACATTCAAGCAGAACTAGGTAGAAACAGAGGGCCAAAATTGAATGCTATGCTTAGATTAGGGGTTTTGCAACCTGAGGTCTATAAACAAAGTCTTCCTGGAAGTAATTGTAAGCATCCTGAAATAAAAAAGCAAGAATATGAAGAAGTAGTTCAGACTGTTAATACAGATTTCTCTCCATATCTGATTTCAGATAACTTAGAACAGCCTATGGGAAGTAGTCATGCATCTCAGGTTTGTTCTGAGACACCTGATGACCTGTTAGATGATGGTGAAATAAAGGAAGATACTAGTTTTGCTGAAAATGACATTAAGGAAAGTTCTGCTGTTTTTAGCAAAAGCGTCCAGAAAGGAGAGCTTAGCAGGAGTCCTAGCCCTTTCACCCATACACATTTGGCTCAGGGTTACCGAAGAGGGGCCAAGAAATTAGAGTCCTCAGAAGAGAACTTATCTAGTGAGGATGAAGAGCTTCCCTGCTTCCAACACTTGTTATTTGGTAAAGTAAACAATATACCTTCTCAGTCTACTAGGCATAGCACCGTTGCTACCGAGTGTCTGTCTAAGAACACAGAGGAGAATTTATTATCATTGAAGAATAGCTTAAATGACTGCAGTAACCAGGTAATATTGGCAAAGGCATCTCAGGAACATCACCTTAGTGAGGAAACAAAATGTTCTGCTAGCTTGTTTTCTTCACAGTGCAGTGAATTGGAAGACTTGACTGCAAATACAAACACCCAGGATCCTTTCTTGATTGGTTCTTCCAAACAAATGAGGCATCAGTCTGAAAGCCAGGGAGTTGGTCTGAGTGACAAGGAATTGGTTTCAGATGATGAAGAAAGAGGAACGGGCTTGGAAGAAAATAATCAAGAAGAGCAAAGCATGGATTCAAACTTAGGTGAAGCAGCATCTGGGTGTGAGAGTGAAACAAGCGTCTCTGAAGACTGCTCAGGGCTATCCTCTCAGAGTGACATTTTAACCACTCAGCAGAGGGATACCATGCAACATAACCTGATAAAGCTCCAGCAGGAAATGGCTGAACTAGAAGCTGTGTTAGAACAGCATGGGAGCCAGCCTTCTAACAGCTACCCTTCCATCATAAGTGACTCTTCTGCCCTTGAGGACCTGCGAAATCCAGAACAAAGCACATCAGAAAAAGCAGTATTAACTTCACAGAAAAGTAGTGAATACCCTATAAGCCAGAATCCAGAAGGCCTTTCTGCTGACAAGTTTGAGGTGTCTGCAGATAGTTCTACCAGTAAAAATAAAGAACCAGGAGTGGAAAGGTCATCCCCTTCTAAATGCCCATCATTAGATGATAGGTGGTACATGCACAGTTGCTCTGGGAGTCTTCAGAATAGAAACTACCCATCTCAAGAGGAGCTCATTAAGGTTGTTGATGTGGAGGAGCAACAGCTGGAAGAGTCTGGGCCACACGATTTGACGGAAACATCTTACTTGCCAAGGCAAGATCTAGAGGGAACCCCTTACCTGGAATCTGGAATCAGCCTCTTCTCTGATGACCCTGAATCTGATCCTTCTGAAGACAGAGCCCCAGAGTCAGCTCGTGTTGGCAACATACCATCTTCAACCTCTGCATTGAAAGTTCCCCAATTGAAAGTTGCAGAATCTGCCCAGAGTCCAGCTGCTGCTCATACTACTGATACTGCTGGGTATAATGCAATGGAAGAAAGTGTGAGCAGGGAGAAGCCAGAATTGACAGCTTCAACAGAAAGGGTCAACAAAAGAATGTCCATGGTGGTGTCTGGCCTGACCCCAGAAGAATTTATGCTCGTGTACAAGTTTGCCAGAAAACACCACATCACTTTAACTAATCTAATTACTGAAGAGACTACTCATGTTGTTATGAAAACAGATGCTGAGTTTGTGTGTGAACGGACACTGAAATATTTTCTAGGAATTGCGGGAGGAAAATGGGTAGTTAGCTATTTCTGGGTGACCCAGTCTATTAAAGAAAGAAAAATGCTGAATGAGCATGATTTTGAAGTCAGAGGAGATGTGGTCAATGGAAGAAACCACCAAGGTCCAAAGCGAGCAAGAGAATCCCAGGACAGAAAGATCTTCAGGGGGCTAGAAATCTGTTGCTATGGGCCCTTCACCAACATGCCCACAGATCAACTGGAATGGATGGTACAGCTGTGTGGTGCTTCTGTGGTGAAGGAGCTTTCATCATTCACCCTTGGCACAGGTGTCCACCCAATTGTGGTTGTGCAGCCAGATGCCTGGACAGAGGACAATGGCTTCCATGCAATTGGGCAGATGTGTGAGGCACCTGTGGTGACCCGAGAGTGGGTGTTGGACAGTGTAGCACTCTACCAGTGCCAGGAGCTGGACACCTACCTGATACCCCAGATCCCCCACAGCCACTACTGA

BRCA1-wild type amino acid sequence

MDLSALRVEEVQNVINAMQKILECPICLELIKEPVSTKCDHIFCKFCMLKLLNQKKGPSQCPLCKNDITKRSLQESTRFSQLVEELLKIICAFQLDTGLEYANSYNFAKKENNSPEHLKDEVSIIQSMGYRNRAKRLLQSEPENPSLQETSLSVQLSNLGTVRTLRTKQRIQPQKTSVYIELGSDSSEDTVNKATYCSVGDQELLQITPQGTRDEISLDSAKKAACEFSETDVTNTEHHQPSNNDLNTTEKRAAERHPEKYQGSSVSNLHVEPCGTNTHASSLQHENSSLLLTKDRMNVEKAEFCNKSKQPGLARSQHNRWAGSKETCNDRRTPSTEKKVDLNADPLCERKEWNKQKLPCSENPRDTEDVPWITLNSSIQKVNEWFSRSDELLGSDDSHDGESESNAKVADVLDVLNEVDEYSGSSEKIDLLASDPHEALICKSERVHSKSVESNIEDKIFGKTYRKKASLPNLSHVTENLIIGAFVTEPQIIQERPLTNKLKRKRRPTSGLHPEDFIKKADLAVQKTPEMINQGTNQTEQNGQVMNITNSGHENKTKGDSIQNEKNPNPIESLEKESAFKTKAEPISSSISNMELELNIHNSKAPKKNRLRRKSSTRHIHALELVVSRNLSPPNCTELQIDSCSSSEEIKKKKYNQMPVRHSRNLQLMEGKEPATGAKKSNKPNEQTSKRHDSDTFPELKLTNAPGSFTKCSNTSELKEFVNPSLPREEKEEKLETVKVSNNAEDPKDLMLSGERVLQTERSVESSSISLVPGTDYGTQESISLLEVSTLGKAKTEPNKCVSQCAAFENPKGLIHGCSKDNRNDTEGFKYPLGHEVNHSRETSIEMEESELDAQYLQNTFKVSKRQSFAPFSNPGNAEEECATFSAHSGSLKKQSPKVTFECEQKEENQGKNESNIKPVQTVNITAGFPVVGQKDKPVDNAKCSIKGGSRFCLSSQFRGNETGLITPNKHGLLQNPYRIPPLFPIKSFVKTKCKKNLLEENFEEHSMSPEREMGNENIPSTVSTISRNNIRENVFKEASSSNINEVGSSTNEVGSSINEIGSSDENIQAELGRNRGPKLNAMLRLGVLQPEVYKQSLPGSNCKHPEIKKQEYEEVVQTVNTDFSPYLISDNLEQPMGSSHASQVCSETPDDLLDDGEIKEDTSFAENDIKESSAVFSKSVQKGELSRSPSPFTHTHLAQGYRRGAKKLESSEENLSSEDEELPCFQHLLFGKVNNIPSQSTRHSTVATECLSKNTEENLLSLKNSLNDCSNQVILAKASQEHHLSEETKCSASLFSSQCSELEDLTANTNTQDPFLIGSSKQMRHQSESQGVGLSDKELVSDDEERGTGLEENNQEEQSMDSNLGEAASGCESETSVSEDCSGLSSQSDILTTQQRDTMQHNLIKLQQEMAELEAVLEQHGSQPSNSYPSIISDSSALEDLRNPEQSTSEKAVLTSQKSSEYPISQNPEGLSADKFEVSADSSTSKNKEPGVERSSPSKCPSLDDRWYMHSCSGSLQNRNYPSQEELIKVVDVEEQQLEESGPHDLTETSYLPRQDLEGTPYLESGISLFSDDPESDPSEDRAPESARVGNIPSSTSALKVPQLKVAESAQSPAAAHTTDTAGYNAMEESVSREKPELTASTERVNKRMSMVVSGLTPEEFMLVYKFARKHHITLTNLITEETTHVVMKTDAEFVCERTLKYFLGIAGGKWVVSYFWVTQSIKERKMLNEHDFEVRGDVVNGRNHQGPKRARESQDRKIFRGLEICCYGPFTNMPTDQLEWMVQLCGASVVKELSSFTLGTGVHPIVVVQPDAWTEDNGFHAIGQMCEAPVVTREWVLDSVALYQCQELDTYLIPQIPHSHY*

BRCA1-coding sequence of exon18 skipping

ATGGATTTATCTGCTCTTCGCGTTGAAGAAGTACAAAATGTCATTAATGCTATGCAGAAAATCTTAGAGTGTCCCATCTGTCTGGAGTTGATCAAGGAACCTGTCTCCACAAAGTGTGACCACATATTTTGCAAATTTTGCATGCTGAAACTTCTCAACCAGAAGAAAGGGCCTTCACAGTGTCCTTTATGTAAGAATGATATAACCAAAAGGAGCCTACAAGAAAGTACGAGATTTAGTCAACTTGTTGAAGAGCTATTGAAAATCATTTGTGCTTTTCAGCTTGACACAGGTTTGGAGTATGCAAACAGCTATAATTTTGCAAAAAAGGAAAATAACTCTCCTGAACATCTAAAAGATGAAGTTTCTATCATCCAAAGTATGGGCTACAGAAACCGTGCCAAAAGACTTCTACAGAGTGAACCCGAAAATCCTTCCTTGCAGGAAACCAGTCTCAGTGTCCAACTCTCTAACCTTGGAACTGTGAGAACTCTGAGGACAAAGCAGCGGATACAACCTCAAAAGACGTCTGTCTACATTGAATTGGGATCTGATTCTTCTGAAGATACCGTTAATAAGGCAACTTATTGCAGTGTGGGAGATCAAGAATTGTTACAAATCACCCCTCAAGGAACCAGGGATGAAATCAGTTTGGATTCTGCAAAAAAGGCTGCTTGTGAATTTTCTGAGACGGATGTAACAAATACTGAACATCATCAACCCAGTAATAATGATTTGAACACCACTGAGAAGCGTGCAGCTGAGAGGCATCCAGAAAAGTATCAGGGTAGTTCTGTTTCAAACTTGCATGTGGAGCCATGTGGCACAAATACTCATGCCAGCTCATTACAGCATGAGAACAGCAGTTTATTACTCACTAAAGACAGAATGAATGTAGAAAAGGCTGAATTCTGTAATAAAAGCAAACAGCCTGGCTTAGCAAGGAGCCAACATAACAGATGGGCTGGAAGTAAGGAAACATGTAATGATAGGCGGACTCCCAGCACAGAAAAAAAGGTAGATCTGAATGCTGATCCCCTGTGTGAGAGAAAAGAATGGAATAAGCAGAAACTGCCATGCTCAGAGAATCCTAGAGATACTGAAGATGTTCCTTGGATAACACTAAATAGCAGCATTCAGAAAGTTAATGAGTGGTTTTCCAGAAGTGATGAACTGTTAGGTTCTGATGACTCACATGATGGGGAGTCTGAATCAAATGCCAAAGTAGCTGATGTATTGGACGTTCTAAATGAGGTAGATGAATATTCTGGTTCTTCAGAGAAAATAGACTTACTGGCCAGTGATCCTCATGAGGCTTTAATATGTAAAAGTGAAAGAGTTCACTCCAAATCAGTAGAGAGTAATATTGAAGACAAAATATTTGGGAAAACCTATCGGAAGAAGGCAAGCCTCCCCAACTTAAGCCATGTAACTGAAAATCTAATTATAGGAGCATTTGTTACTGAGCCACAGATAATACAAGAGCGTCCCCTCACAAATAAATTAAAGCGTAAAAGGAGACCTACATCAGGCCTTCATCCTGAGGATTTTATCAAGAAAGCAGATTTGGCAGTTCAAAAGACTCCTGAAATGATAAATCAGGGAACTAACCAAACGGAGCAGAATGGTCAAGTGATGAATATTACTAATAGTGGTCATGAGAATAAAACAAAAGGTGATTCTATTCAGAATGAGAAAAATCCTAACCCAATAGAATCACTCGAAAAAGAATCTGCTTTCAAAACGAAAGCTGAACCTATAAGCAGCAGTATAAGCAATATGGAACTCGAATTAAATATCCACAATTCAAAAGCACCTAAAAAGAATAGGCTGAGGAGGAAGTCTTCTACCAGGCATATTCATGCGCTTGAACTAGTAGTCAGTAGAAATCTAAGCCCACCTAATTGTACTGAATTGCAAATTGATAGTTGTTCTAGCAGTGAAGAGATAAAGAAAAAAAAGTACAACCAAATGCCAGTCAGGCACAGCAGAAACCTACAACTCATGGAAGGTAAAGAACCTGCAACTGGAGCCAAGAAGAGTAACAAGCCAAATGAACAGACAAGTAAAAGACATGACAGCGATACTTTCCCAGAGCTGAAGTTAACAAATGCACCTGGTTCTTTTACTAAGTGTTCAAATACCAGTGAACTTAAAGAATTTGTCAATCCTAGCCTTCCAAGAGAAGAAAAAGAAGAGAAACTAGAAACAGTTAAAGTGTCTAATAATGCTGAAGACCCCAAAGATCTCATGTTAAGTGGAGAAAGGGTTTTGCAAACTGAAAGATCTGTAGAGAGTAGCAGTATTTCATTGGTACCTGGTACTGATTATGGCACTCAGGAAAGTATCTCGTTACTGGAAGTTAGCACTCTAGGGAAGGCAAAAACAGAACCAAATAAATGTGTGAGTCAGTGTGCAGCATTTGAAAACCCCAAGGGACTAATTCATGGTTGTTCCAAAGATAATAGAAATGACACAGAAGGCTTTAAGTATCCATTGGGACATGAAGTTAACCACAGTCGGGAAACAAGCATAGAAATGGAAGAAAGTGAACTTGATGCTCAGTATTTGCAGAATACATTCAAGGTTTCAAAGCGCCAGTCATTTGCTCCGTTTTCAAATCCAGGAAATGCAGAAGAGGAATGTGCAACATTCTCTGCCCACTCTGGGTCCTTAAAGAAACAAAGTCCAAAAGTCACTTTTGAATGTGAACAAAAGGAAGAAAATCAAGGAAAGAATGAGTCTAATATCAAGCCTGTACAGACAGTTAATATCACTGCAGGCTTTCCTGTGGTTGGTCAGAAAGATAAGCCAGTTGATAATGCCAAATGTAGTATCAAAGGAGGCTCTAGGTTTTGTCTATCATCTCAGTTCAGAGGCAACGAAACTGGACTCATTACTCCAAATAAACATGGACTTTTACAAAACCCATATCGTATACCACCACTTTTTCCCATCAAGTCATTTGTTAAAACTAAATGTAAGAAAAATCTGCTAGAGGAAAACTTTGAGGAACATTCAATGTCACCTGAAAGAGAAATGGGAAATGAGAACATTCCAAGTACAGTGAGCACAATTAGCCGTAATAACATTAGAGAAAATGTTTTTAAAGAAGCCAGCTCAAGCAATATTAATGAAGTAGGTTCCAGTACTAATGAAGTGGGCTCCAGTATTAATGAAATAGGTTCCAGTGATGAAAACATTCAAGCAGAACTAGGTAGAAACAGAGGGCCAAAATTGAATGCTATGCTTAGATTAGGGGTTTTGCAACCTGAGGTCTATAAACAAAGTCTTCCTGGAAGTAATTGTAAGCATCCTGAAATAAAAAAGCAAGAATATGAAGAAGTAGTTCAGACTGTTAATACAGATTTCTCTCCATATCTGATTTCAGATAACTTAGAACAGCCTATGGGAAGTAGTCATGCATCTCAGGTTTGTTCTGAGACACCTGATGACCTGTTAGATGATGGTGAAATAAAGGAAGATACTAGTTTTGCTGAAAATGACATTAAGGAAAGTTCTGCTGTTTTTAGCAAAAGCGTCCAGAAAGGAGAGCTTAGCAGGAGTCCTAGCCCTTTCACCCATACACATTTGGCTCAGGGTTACCGAAGAGGGGCCAAGAAATTAGAGTCCTCAGAAGAGAACTTATCTAGTGAGGATGAAGAGCTTCCCTGCTTCCAACACTTGTTATTTGGTAAAGTAAACAATATACCTTCTCAGTCTACTAGGCATAGCACCGTTGCTACCGAGTGTCTGTCTAAGAACACAGAGGAGAATTTATTATCATTGAAGAATAGCTTAAATGACTGCAGTAACCAGGTAATATTGGCAAAGGCATCTCAGGAACATCACCTTAGTGAGGAAACAAAATGTTCTGCTAGCTTGTTTTCTTCACAGTGCAGTGAATTGGAAGACTTGACTGCAAATACAAACACCCAGGATCCTTTCTTGATTGGTTCTTCCAAACAAATGAGGCATCAGTCTGAAAGCCAGGGAGTTGGTCTGAGTGACAAGGAATTGGTTTCAGATGATGAAGAAAGAGGAACGGGCTTGGAAGAAAATAATCAAGAAGAGCAAAGCATGGATTCAAACTTAGGTGAAGCAGCATCTGGGTGTGAGAGTGAAACAAGCGTCTCTGAAGACTGCTCAGGGCTATCCTCTCAGAGTGACATTTTAACCACTCAGCAGAGGGATACCATGCAACATAACCTGATAAAGCTCCAGCAGGAAATGGCTGAACTAGAAGCTGTGTTAGAACAGCATGGGAGCCAGCCTTCTAACAGCTACCCTTCCATCATAAGTGACTCTTCTGCCCTTGAGGACCTGCGAAATCCAGAACAAAGCACATCAGAAAAAGCAGTATTAACTTCACAGAAAAGTAGTGAATACCCTATAAGCCAGAATCCAGAAGGCCTTTCTGCTGACAAGTTTGAGGTGTCTGCAGATAGTTCTACCAGTAAAAATAAAGAACCAGGAGTGGAAAGGTCATCCCCTTCTAAATGCCCATCATTAGATGATAGGTGGTACATGCACAGTTGCTCTGGGAGTCTTCAGAATAGAAACTACCCATCTCAAGAGGAGCTCATTAAGGTTGTTGATGTGGAGGAGCAACAGCTGGAAGAGTCTGGGCCACACGATTTGACGGAAACATCTTACTTGCCAAGGCAAGATCTAGAGGGAACCCCTTACCTGGAATCTGGAATCAGCCTCTTCTCTGATGACCCTGAATCTGATCCTTCTGAAGACAGAGCCCCAGAGTCAGCTCGTGTTGGCAACATACCATCTTCAACCTCTGCATTGAAAGTTCCCCAATTGAAAGTTGCAGAATCTGCCCAGAGTCCAGCTGCTGCTCATACTACTGATACTGCTGGGTATAATGCAATGGAAGAAAGTGTGAGCAGGGAGAAGCCAGAATTGACAGCTTCAACAGAAAGGGTCAACAAAAGAATGTCCATGGTGGTGTCTGGCCTGACCCCAGAAGAATTTATGCTCGTGTACAAGTTTGCCAGAAAACACCACATCACTTTAACTAATCTAATTACTGAAGAGACTACTCATGTTGTTATGAAAACAGATGCTGAGTTTGTGTGTGAACGGACACTGAAATATTTTCTAGGAATTGCGGGAGGAAAATGGGTAGTTAGCTATTTCTCATGATTTTGAAGTCAGAGGAGATGTGGTCAATGGAAGAAACCACCAAGGTCCAAAGCGAGCAAGAGAATCCCAGGACAGAAAGATCTTCAGGGGGCTAGAAATCTGTTGCTATGGGCCCTTCACCAACATGCCCACAGATCAACTGGAATGGATGGTACAGCTGTGTGGTGCTTCTGTGGTGAAGGAGCTTTCATCATTCACCCTTGGCACAGGTGTCCACCCAATTGTGGTTGTGCAGCCAGATGCCTGGACAGAGGACAATGGCTTCCATGCAATTGGGCAGATGTGTGAGGCACCTGTGGTGACCCGAGAGTGGGTGTTGGACAGTGTAGCACTCTACCAGTGCCAGGAGCTGGACACCTACCTGATACCCCAGATCCCCCACAGCCACTACTGA

BRCA1- amino acid sequence of exon18 skipping

MDLSALRVEEVQNVINAMQKILECPICLELIKEPVSTKCDHIFCKFCMLKLLNQKKGPSQCPLCKNDITKRSLQESTRFSQLVEELLKIICAFQLDTGLEYANSYNFAKKENNSPEHLKDEVSIIQSMGYRNRAKRLLQSEPENPSLQETSLSVQLSNLGTVRTLRTKQRIQPQKTSVYIELGSDSSEDTVNKATYCSVGDQELLQITPQGTRDEISLDSAKKAACEFSETDVTNTEHHQPSNNDLNTTEKRAAERHPEKYQGSSVSNLHVEPCGTNTHASSLQHENSSLLLTKDRMNVEKAEFCNKSKQPGLARSQHNRWAGSKETCNDRRTPSTEKKVDLNADPLCERKEWNKQKLPCSENPRDTEDVPWITLNSSIQKVNEWFSRSDELLGSDDSHDGESESNAKVADVLDVLNEVDEYSGSSEKIDLLASDPHEALICKSERVHSKSVESNIEDKIFGKTYRKKASLPNLSHVTENLIIGAFVTEPQIIQERPLTNKLKRKRRPTSGLHPEDFIKKADLAVQKTPEMINQGTNQTEQNGQVMNITNSGHENKTKGDSIQNEKNPNPIESLEKESAFKTKAEPISSSISNMELELNIHNSKAPKKNRLRRKSSTRHIHALELVVSRNLSPPNCTELQIDSCSSSEEIKKKKYNQMPVRHSRNLQLMEGKEPATGAKKSNKPNEQTSKRHDSDTFPELKLTNAPGSFTKCSNTSELKEFVNPSLPREEKEEKLETVKVSNNAEDPKDLMLSGERVLQTERSVESSSISLVPGTDYGTQESISLLEVSTLGKAKTEPNKCVSQCAAFENPKGLIHGCSKDNRNDTEGFKYPLGHEVNHSRETSIEMEESELDAQYLQNTFKVSKRQSFAPFSNPGNAEEECATFSAHSGSLKKQSPKVTFECEQKEENQGKNESNIKPVQTVNITAGFPVVGQKDKPVDNAKCSIKGGSRFCLSSQFRGNETGLITPNKHGLLQNPYRIPPLFPIKSFVKTKCKKNLLEENFEEHSMSPEREMGNENIPSTVSTISRNNIRENVFKEASSSNINEVGSSTNEVGSSINEIGSSDENIQAELGRNRGPKLNAMLRLGVLQPEVYKQSLPGSNCKHPEIKKQEYEEVVQTVNTDFSPYLISDNLEQPMGSSHASQVCSETPDDLLDDGEIKEDTSFAENDIKESSAVFSKSVQKGELSRSPSPFTHTHLAQGYRRGAKKLESSEENLSSEDEELPCFQHLLFGKVNNIPSQSTRHSTVATECLSKNTEENLLSLKNSLNDCSNQVILAKASQEHHLSEETKCSASLFSSQCSELEDLTANTNTQDPFLIGSSKQMRHQSESQGVGLSDKELVSDDEERGTGLEENNQEEQSMDSNLGEAASGCESETSVSEDCSGLSSQSDILTTQQRDTMQHNLIKLQQEMAELEAVLEQHGSQPSNSYPSIISDSSALEDLRNPEQSTSEKAVLTSQKSSEYPISQNPEGLSADKFEVSADSSTSKNKEPGVERSSPSKCPSLDDRWYMHSCSGSLQNRNYPSQEELIKVVDVEEQQLEESGPHDLTETSYLPRQDLEGTPYLESGISLFSDDPESDPSEDRAPESARVGNIPSSTSALKVPQLKVAESAQSPAAAHTTDTAGYNAMEESVSREKPELTASTERVNKRMSMVVSGLTPEEFMLVYKFARKHHITLTNLITEETTHVVMKTDAEFVCERTLKYFLGIAGGKWVVSYFS*
