# Supplementary material for: Case Report: Functional validation of a rare variant BRCA1 c.5193 + 2dupT in a family with cancer history
Source: Front Oncol. 2025 Sep 30;15:1623700. doi: 10.3389/fonc.2025.1623700 (PMC12518100; doi:10.3389/fonc.2025.1623700)
Supplement: Supplementary file 1 [file DataSheet1.zip › Supplementary Material/BRCA1-the coding sequence and amino acid sequences.pdf]

BRCA1-wild type coding sequence

ATGGATTTATCTGCTCTTCGCGTTGAAGAAGTACAAAATGTCATTAATGCTATGC
AGAAAATCTTAGAGTGTCCCATCTGTCTGGAGTTGATCAAGGAACCTGTCTCCAC
AAAGTGTGACCACATATTTTGCAAATTTTGCATGCTGAAACTTCTCAACCAGAAG
AAAGGGCCTTCACAGTGTCCTTTATGTAAGAATGATATAACCAAAAGGAGCCTAC
AAGAAAGTACGAGATTTAGTCAACTTGTTGAAGAGCTATTGAAAATCATTTGTGC
TTTTCAGCTTGACACAGGTTTGGAGTATGCAAACAGCTATAATTTTGCAAAAAAG
GAAAATAACTCTCCTGAACATCTAAAAGATGAAGTTTCTATCATCCAAAGTATGG
GCTACAGAAACCGTGCCAAAAGACTTCTACAGAGTGAACCCGAAAATCCTTCCTT
GCAGGAAACCAGTCTCAGTGTCCAACTCTCTAACCTTGGAACTGTGAGAACTCTG
AGGACAAAGCAGCGGATACAACCTCAAAAGACGTCTGTCTACATTGAATTGGGA
TCTGATTCTTCTGAAGATACCGTTAATAAGGCAACTTATTGCAGTGTGGGAGATC
AAGAATTGTTACAAATCACCCCTCAAGGAACCAGGGATGAAATCAGTTTGGATTC
TGCAAAAAAGGCTGCTTGTGAATTTTCTGAGACGGATGTAACAAATACTGAACAT
CATCAACCCAGTAATAATGATTTGAACACCACTGAGAAGCGTGCAGCTGAGAGG
CATCCAGAAAAGTATCAGGGTAGTTCTGTTTCAAACTTGCATGTGGAGCCATGTG
GCACAAATACTCATGCCAGCTCATTACAGCATGAGAACAGCAGTTTATTACTCAC
TAAAGACAGAATGAATGTAGAAAAGGCTGAATTCTGTAATAAAAGCAAACAGCC
TGGCTTAGCAAGGAGCCAACATAACAGATGGGCTGGAAGTAAGGAAACATGTAA
TGATAGGCGGACTCCCAGCACAGAAAAAAAGGTAGATCTGAATGCTGATCCCCT
GTGTGAGAGAAAAGAATGGAATAAGCAGAAACTGCCATGCTCAGAGAATCCTAG
AGATACTGAAGATGTTCCTTGGATAACACTAAATAGCAGCATTCAGAAAGTTAAT
GAGTGGTTTTCCAGAAGTGATGAACTGTTAGGTTCTGATGACTCACATGATGGGG
AGTCTGAATCAAATGCCAAAGTAGCTGATGTATTGGACGTTCTAAATGAGGTAGA
TGAATATTCTGGTTCTTCAGAGAAAATAGACTTACTGGCCAGTGATCCTCATGAG
GCTTTAATATGTAAAAGTGAAAGAGTTCACTCCAAATCAGTAGAGAGTAATATTG
AAGACAAAATATTTGGGAAAACCTATCGGAAGAAGGCAAGCCTCCCCAACTTAA
GCCATGTAACTGAAAATCTAATTATAGGAGCATTTGTTACTGAGCCACAGATAAT
ACAAGAGCGTCCCCTCACAAATAAATTAAAGCGTAAAAGGAGACCTACATCAGG
CCTTCATCCTGAGGATTTTATCAAGAAAGCAGATTTGGCAGTTCAAAAGACTCCT
GAAATGATAAATCAGGGAACTAACCAAACGGAGCAGAATGGTCAAGTGATGAAT
ATTACTAATAGTGGTCATGAGAATAAAACAAAAGGTGATTCTATTCAGAATGAG
AAAAATCCTAACCCAATAGAATCACTCGAAAAAGAATCTGCTTTCAAAACGAAA
GCTGAACCTATAAGCAGCAGTATAAGCAATATGGAACTCGAATTAAATATCCAC
AATTCAAAAGCACCTAAAAAGAATAGGCTGAGGAGGAAGTCTTCTACCAGGCAT
ATTCATGCGCTTGAACTAGTAGTCAGTAGAAATCTAAGCCCACCTAATTGTACTG
AATTGCAAATTGATAGTTGTTCTAGCAGTGAAGAGATAAAGAAAAAAAAGTACA
ACCAAATGCCAGTCAGGCACAGCAGAAACCTACAACTCATGGAAGGTAAAGAAC

CTGCAACTGGAGCCAAGAAGAGTAACAAGCCAAATGAACAGACAAGTAAAAGA
CATGACAGCGATACTTTCCCAGAGCTGAAGTTAACAAATGCACCTGGTTCTTTTA
CTAAGTGTTCAAATACCAGTGAACTTAAAGAATTTGTCAATCCTAGCCTTCCAAG
AGAAGAAAAAGAAGAGAAACTAGAAACAGTTAAAGTGTCTAATAATGCTGAAG
ACCCCAAAGATCTCATGTTAAGTGGAGAAAGGGTTTTGCAAACTGAAAGATCTGT
AGAGAGTAGCAGTATTTCATTGGTACCTGGTACTGATTATGGCACTCAGGAAAGT
ATCTCGTTACTGGAAGTTAGCACTCTAGGGAAGGCAAAAACAGAACCAAATAAA
TGTGTGAGTCAGTGTGCAGCATTTGAAAACCCCAAGGGACTAATTCATGGTTGTT
CCAAAGATAATAGAAATGACACAGAAGGCTTTAAGTATCCATTGGGACATGAAG
TTAACCACAGTCGGGAAACAAGCATAGAAATGGAAGAAAGTGAACTTGATGCTC
AGTATTTGCAGAATACATTCAAGGTTTCAAAGCGCCAGTCATTTGCTCCGTTTTCA
AATCCAGGAAATGCAGAAGAGGAATGTGCAACATTCTCTGCCCACTCTGGGTCCT
TAAAGAAACAAAGTCCAAAAGTCACTTTTGAATGTGAACAAAAGGAAGAAAATC
AAGGAAAGAATGAGTCTAATATCAAGCCTGTACAGACAGTTAATATCACTGCAG
GCTTTCCTGTGGTTGGTCAGAAAGATAAGCCAGTTGATAATGCCAAATGTAGTAT
CAAAGGAGGCTCTAGGTTTTGTCTATCATCTCAGTTCAGAGGCAACGAAACTGGA
CTCATTACTCCAAATAAACATGGACTTTTACAAAACCCATATCGTATACCACCAC
TTTTTCCCATCAAGTCATTTGTTAAAACTAAATGTAAGAAAAATCTGCTAGAGGA
AAACTTTGAGGAACATTCAATGTCACCTGAAAGAGAAATGGGAAATGAGAACAT
TCCAAGTACAGTGAGCACAATTAGCCGTAATAACATTAGAGAAAATGTTTTTAAA
GAAGCCAGCTCAAGCAATATTAATGAAGTAGGTTCCAGTACTAATGAAGTGGGC
TCCAGTATTAATGAAATAGGTTCCAGTGATGAAAACATTCAAGCAGAACTAGGTA
GAAACAGAGGGCCAAAATTGAATGCTATGCTTAGATTAGGGGTTTTGCAACCTGA
GGTCTATAAACAAAGTCTTCCTGGAAGTAATTGTAAGCATCCTGAAATAAAAAAG
CAAGAATATGAAGAAGTAGTTCAGACTGTTAATACAGATTTCTCTCCATATCTGA
TTTCAGATAACTTAGAACAGCCTATGGGAAGTAGTCATGCATCTCAGGTTTGTTC
TGAGACACCTGATGACCTGTTAGATGATGGTGAAATAAAGGAAGATACTAGTTTT
GCTGAAAATGACATTAAGGAAAGTTCTGCTGTTTTTAGCAAAAGCGTCCAGAAAG
GAGAGCTTAGCAGGAGTCCTAGCCCTTTCACCCATACACATTTGGCTCAGGGTTA
CCGAAGAGGGGCCAAGAAATTAGAGTCCTCAGAAGAGAACTTATCTAGTGAGGA
TGAAGAGCTTCCCTGCTTCCAACACTTGTTATTTGGTAAAGTAAACAATATACCTT
CTCAGTCTACTAGGCATAGCACCGTTGCTACCGAGTGTCTGTCTAAGAACACAGA
GGAGAATTTATTATCATTGAAGAATAGCTTAAATGACTGCAGTAACCAGGTAATA
TTGGCAAAGGCATCTCAGGAACATCACCTTAGTGAGGAAACAAAATGTTCTGCTA
GCTTGTTTTCTTCACAGTGCAGTGAATTGGAAGACTTGACTGCAAATACAAACAC
CCAGGATCCTTTCTTGATTGGTTCTTCCAAACAAATGAGGCATCAGTCTGAAAGC
CAGGGAGTTGGTCTGAGTGACAAGGAATTGGTTTCAGATGATGAAGAAAGAGGA
ACGGGCTTGGAAGAAAATAATCAAGAAGAGCAAAGCATGGATTCAAACTTAGGT

GAAGCAGCATCTGGGTGTGAGAGTGAAACAAGCGTCTCTGAAGACTGCTCAGGG
CTATCCTCTCAGAGTGACATTTTAACCACTCAGCAGAGGGATACCATGCAACATA
ACCTGATAAAGCTCCAGCAGGAAATGGCTGAACTAGAAGCTGTGTTAGAACAGC
ATGGGAGCCAGCCTTCTAACAGCTACCCTTCCATCATAAGTGACTCTTCTGCCCTT
GAGGACCTGCGAAATCCAGAACAAAGCACATCAGAAAAAGCAGTATTAACTTCA
CAGAAAAGTAGTGAATACCCTATAAGCCAGAATCCAGAAGGCCTTTCTGCTGAC
AAGTTTGAGGTGTCTGCAGATAGTTCTACCAGTAAAAATAAAGAACCAGGAGTG
GAAAGGTCATCCCCTTCTAAATGCCCATCATTAGATGATAGGTGGTACATGCACA
GTTGCTCTGGGAGTCTTCAGAATAGAAACTACCCATCTCAAGAGGAGCTCATTAA
GGTTGTTGATGTGGAGGAGCAACAGCTGGAAGAGTCTGGGCCACACGATTTGAC
GGAAACATCTTACTTGCCAAGGCAAGATCTAGAGGGAACCCCTTACCTGGAATCT
GGAATCAGCCTCTTCTCTGATGACCCTGAATCTGATCCTTCTGAAGACAGAGCCC
CAGAGTCAGCTCGTGTTGGCAACATACCATCTTCAACCTCTGCATTGAAAGTTCC
CCAATTGAAAGTTGCAGAATCTGCCCAGAGTCCAGCTGCTGCTCATACTACTGAT
ACTGCTGGGTATAATGCAATGGAAGAAAGTGTGAGCAGGGAGAAGCCAGAATTG
ACAGCTTCAACAGAAAGGGTCAACAAAAGAATGTCCATGGTGGTGTCTGGCCTG
ACCCCAGAAGAATTTATGCTCGTGTACAAGTTTGCCAGAAAACACCACATCACTT
TAACTAATCTAATTACTGAAGAGACTACTCATGTTGTTATGAAAACAGATGCTGA
GTTTGTGTGTGAACGGACACTGAAATATTTTCTAGGAATTGCGGGAGGAAAATGG
GTAGTTAGCTATTTCTGGGTGACCCAGTCTATTAAAGAAAGAAAAATGCTGAATG
AGCATGATTTTGAAGTCAGAGGAGATGTGGTCAATGGAAGAAACCACCAAGGTC
CAAAGCGAGCAAGAGAATCCCAGGACAGAAAGATCTTCAGGGGGCTAGAAATCT
GTTGCTATGGGCCCTTCACCAACATGCCCACAGATCAACTGGAATGGATGGTACA
GCTGTGTGGTGCTTCTGTGGTGAAGGAGCTTTCATCATTCACCCTTGGCACAGGT
GTCCACCCAATTGTGGTTGTGCAGCCAGATGCCTGGACAGAGGACAATGGCTTCC
ATGCAATTGGGCAGATGTGTGAGGCACCTGTGGTGACCCGAGAGTGGGTGTTGG
ACAGTGTAGCACTCTACCAGTGCCAGGAGCTGGACACCTACCTGATACCCCAGAT
CCCCCACAGCCACTACTGA

BRCA1-wild type amino acid sequence

MDLSALRVEEVQNVINAMQKILECPICLELIKEPVSTKCDHIFCKFCMLKLLNQKKGP
SQCPLCKNDITKRSLQESTRFSQLVEELLKIICAFQLDTGLEYANSYNFAKKENNSPEH
LKDEVSIIQSMGYRNRAKRLLQSEPENPSLQETSLSVQLSNLGTVRTLRTKQRIQPQKT
SVYIELGSDSSEDTVNKATYCSVGDQELLQITPQGTRDEISLDSAKKAACEFSETDVT
NTEHHQPSNNDLNTTEKRAAERHPEKYQGSSVSNLHVEPCGTNTHASSLQHENSSLL
LTKDRMNVEKAEFCNKSKQPGLARSQHNRWAGSKETCNDRRTPSTEKKVDLNADP
LCERKEWNKQKLPCSENPRDTEDVPWITLNSSIQKVNEWFSRSDELLGSDDSHDGES
ESNAKVADVLDVLNEVDEYSGSSEKIDLLASDPHEALICKSERVHSKSVESNIEDKIFG

KTYRKKASLPNLSHVTENLIIGAFVTEPQIIQERPLTNKLKRKRRPTSGLHPEDFIKKA
DLAVQKTPEMINQGTNQTEQNGQVMNITNSGHENKTKGDSIQNEKNPNPIESLEKES
AFKTKAEPISSSISNMELELNIHNSKAPKKNRLRRKSSTRHIHALELVVSRNLSPPNCTE
LQIDSCSSSEEIKKKKYNQMPVRHSRNLQLMEGKEPATGAKKSNKPNEQTSKRHDSD
TFPELKLTNAPGSFTKCSNTSELKEFVNPSLPREEKEEKLETVKVSNNAEDPKDLMLS
GERVLQTERSVESSSISLVPGTDYGTQESISLLEVSTLGKAKTEPNKCVSQCAAFENPK
GLIHGCSKDNRNDTEGFKYPLGHEVNHSRETSIEMEESELDAQYLQNTFKVSKRQSF
APFSNPGNAEEECATFSAHSGSLKKQSPKVTFECEQKEENQGKNESNIKPVQTVNITA
GFPVVGQKDKPVDNAKCSIKGGSRFCLSSQFRGNETGLITPNKHGLLQNPYRIPPLFPI
KSFVKTKCKKNLLEENFEEHSMSPEREMGNENIPSTVSTISRNNIRENVFKEASSSNIN
EVGSSTNEVGSSINEIGSSDENIQAELGRNRGPKLNAMLRLGVLQPEVYKQSLPGSNC
KHPEIKKQEYEEVVQTVNTDFSPYLISDNLEQPMGSSHASQVCSETPDDLLDDGEIKE
DTSFAENDIKESSAVFSKSVQKGELSRSPSPFTHTHLAQGYRRGAKKLESSEENLSSED
EELPCFQHLLFGKVNNIPSQSTRHSTVATECLSKNTEENLLSLKNSLNDCSNQVILAK
ASQEHHLSEETKCSASLFSSQCSELEDLTANTNTQDPFLIGSSKQMRHQSESQGVGLS
DKELVSDDEERGTGLEENNQEEQSMDSNLGEAASGCESETSVSEDCSGLSSQSDILTT
QQRDTMQHNLIKLQQEMAELEAVLEQHGSQPSNSYPSIISDSSALEDLRNPEQSTSEK
AVLTSQKSSEYPISQNPEGLSADKFEVSADSSTSKNKEPGVERSSPSKCPSLDDRWYM
HSCSGSLQNRNYPSQEELIKVVDVEEQQLEESGPHDLTETSYLPRQDLEGTPYLESGIS
LFSDDPESDPSEDRAPESARVGNIPSSTSALKVPQLKVAESAQSPAAAHTTDTAGYNA
MEESVSREKPELTASTERVNKRMSMVVSGLTPEEFMLVYKFARKHHITLTNLITEETT
HVVMKTDAEFVCERTLKYFLGIAGGKWVVSYFWVTQSIKERKMLNEHDFEVRGDV
VNGRNHQGPKRARESQDRKIFRGLEICCYGPFTNMPTDQLEWMVQLCGASVVKELS
SFTLGTGVHPIVVVQPDAWTEDNGFHAIGQMCEAPVVTREWVLDSVALYQCQELDT
YLIPQIPHSHY*

BRCA1-coding sequence of exon18 skipping

ATGGATTTATCTGCTCTTCGCGTTGAAGAAGTACAAAATGTCATTAATGCTATGC
AGAAAATCTTAGAGTGTCCCATCTGTCTGGAGTTGATCAAGGAACCTGTCTCCAC
AAAGTGTGACCACATATTTTGCAAATTTTGCATGCTGAAACTTCTCAACCAGAAG
AAAGGGCCTTCACAGTGTCCTTTATGTAAGAATGATATAACCAAAAGGAGCCTAC
AAGAAAGTACGAGATTTAGTCAACTTGTTGAAGAGCTATTGAAAATCATTTGTGC
TTTTCAGCTTGACACAGGTTTGGAGTATGCAAACAGCTATAATTTTGCAAAAAAG
GAAAATAACTCTCCTGAACATCTAAAAGATGAAGTTTCTATCATCCAAAGTATGG
GCTACAGAAACCGTGCCAAAAGACTTCTACAGAGTGAACCCGAAAATCCTTCCTT
GCAGGAAACCAGTCTCAGTGTCCAACTCTCTAACCTTGGAACTGTGAGAACTCTG
AGGACAAAGCAGCGGATACAACCTCAAAAGACGTCTGTCTACATTGAATTGGGA
TCTGATTCTTCTGAAGATACCGTTAATAAGGCAACTTATTGCAGTGTGGGAGATC

AAGAATTGTTACAAATCACCCCTCAAGGAACCAGGGATGAAATCAGTTTGGATTC
TGCAAAAAAGGCTGCTTGTGAATTTTCTGAGACGGATGTAACAAATACTGAACAT
CATCAACCCAGTAATAATGATTTGAACACCACTGAGAAGCGTGCAGCTGAGAGG
CATCCAGAAAAGTATCAGGGTAGTTCTGTTTCAAACTTGCATGTGGAGCCATGTG
GCACAAATACTCATGCCAGCTCATTACAGCATGAGAACAGCAGTTTATTACTCAC
TAAAGACAGAATGAATGTAGAAAAGGCTGAATTCTGTAATAAAAGCAAACAGCC
TGGCTTAGCAAGGAGCCAACATAACAGATGGGCTGGAAGTAAGGAAACATGTAA
TGATAGGCGGACTCCCAGCACAGAAAAAAAGGTAGATCTGAATGCTGATCCCCT
GTGTGAGAGAAAAGAATGGAATAAGCAGAAACTGCCATGCTCAGAGAATCCTAG
AGATACTGAAGATGTTCCTTGGATAACACTAAATAGCAGCATTCAGAAAGTTAAT
GAGTGGTTTTCCAGAAGTGATGAACTGTTAGGTTCTGATGACTCACATGATGGGG
AGTCTGAATCAAATGCCAAAGTAGCTGATGTATTGGACGTTCTAAATGAGGTAGA
TGAATATTCTGGTTCTTCAGAGAAAATAGACTTACTGGCCAGTGATCCTCATGAG
GCTTTAATATGTAAAAGTGAAAGAGTTCACTCCAAATCAGTAGAGAGTAATATTG
AAGACAAAATATTTGGGAAAACCTATCGGAAGAAGGCAAGCCTCCCCAACTTAA
GCCATGTAACTGAAAATCTAATTATAGGAGCATTTGTTACTGAGCCACAGATAAT
ACAAGAGCGTCCCCTCACAAATAAATTAAAGCGTAAAAGGAGACCTACATCAGG
CCTTCATCCTGAGGATTTTATCAAGAAAGCAGATTTGGCAGTTCAAAAGACTCCT
GAAATGATAAATCAGGGAACTAACCAAACGGAGCAGAATGGTCAAGTGATGAAT
ATTACTAATAGTGGTCATGAGAATAAAACAAAAGGTGATTCTATTCAGAATGAG
AAAAATCCTAACCCAATAGAATCACTCGAAAAAGAATCTGCTTTCAAAACGAAA
GCTGAACCTATAAGCAGCAGTATAAGCAATATGGAACTCGAATTAAATATCCAC
AATTCAAAAGCACCTAAAAAGAATAGGCTGAGGAGGAAGTCTTCTACCAGGCAT
ATTCATGCGCTTGAACTAGTAGTCAGTAGAAATCTAAGCCCACCTAATTGTACTG
AATTGCAAATTGATAGTTGTTCTAGCAGTGAAGAGATAAAGAAAAAAAAGTACA
ACCAAATGCCAGTCAGGCACAGCAGAAACCTACAACTCATGGAAGGTAAAGAAC
CTGCAACTGGAGCCAAGAAGAGTAACAAGCCAAATGAACAGACAAGTAAAAGA
CATGACAGCGATACTTTCCCAGAGCTGAAGTTAACAAATGCACCTGGTTCTTTTA
CTAAGTGTTCAAATACCAGTGAACTTAAAGAATTTGTCAATCCTAGCCTTCCAAG
AGAAGAAAAAGAAGAGAAACTAGAAACAGTTAAAGTGTCTAATAATGCTGAAG
ACCCCAAAGATCTCATGTTAAGTGGAGAAAGGGTTTTGCAAACTGAAAGATCTGT
AGAGAGTAGCAGTATTTCATTGGTACCTGGTACTGATTATGGCACTCAGGAAAGT
ATCTCGTTACTGGAAGTTAGCACTCTAGGGAAGGCAAAAACAGAACCAAATAAA
TGTGTGAGTCAGTGTGCAGCATTTGAAAACCCCAAGGGACTAATTCATGGTTGTT
CCAAAGATAATAGAAATGACACAGAAGGCTTTAAGTATCCATTGGGACATGAAG
TTAACCACAGTCGGGAAACAAGCATAGAAATGGAAGAAAGTGAACTTGATGCTC
AGTATTTGCAGAATACATTCAAGGTTTCAAAGCGCCAGTCATTTGCTCCGTTTTCA
AATCCAGGAAATGCAGAAGAGGAATGTGCAACATTCTCTGCCCACTCTGGGTCCT

TAAAGAAACAAAGTCCAAAAGTCACTTTTGAATGTGAACAAAAGGAAGAAAATC
AAGGAAAGAATGAGTCTAATATCAAGCCTGTACAGACAGTTAATATCACTGCAG
GCTTTCCTGTGGTTGGTCAGAAAGATAAGCCAGTTGATAATGCCAAATGTAGTAT
CAAAGGAGGCTCTAGGTTTTGTCTATCATCTCAGTTCAGAGGCAACGAAACTGGA
CTCATTACTCCAAATAAACATGGACTTTTACAAAACCCATATCGTATACCACCAC
TTTTTCCCATCAAGTCATTTGTTAAAACTAAATGTAAGAAAAATCTGCTAGAGGA
AAACTTTGAGGAACATTCAATGTCACCTGAAAGAGAAATGGGAAATGAGAACAT
TCCAAGTACAGTGAGCACAATTAGCCGTAATAACATTAGAGAAAATGTTTTTAAA
GAAGCCAGCTCAAGCAATATTAATGAAGTAGGTTCCAGTACTAATGAAGTGGGC
TCCAGTATTAATGAAATAGGTTCCAGTGATGAAAACATTCAAGCAGAACTAGGTA
GAAACAGAGGGCCAAAATTGAATGCTATGCTTAGATTAGGGGTTTTGCAACCTGA
GGTCTATAAACAAAGTCTTCCTGGAAGTAATTGTAAGCATCCTGAAATAAAAAAG
CAAGAATATGAAGAAGTAGTTCAGACTGTTAATACAGATTTCTCTCCATATCTGA
TTTCAGATAACTTAGAACAGCCTATGGGAAGTAGTCATGCATCTCAGGTTTGTTC
TGAGACACCTGATGACCTGTTAGATGATGGTGAAATAAAGGAAGATACTAGTTTT
GCTGAAAATGACATTAAGGAAAGTTCTGCTGTTTTTAGCAAAAGCGTCCAGAAAG
GAGAGCTTAGCAGGAGTCCTAGCCCTTTCACCCATACACATTTGGCTCAGGGTTA
CCGAAGAGGGGCCAAGAAATTAGAGTCCTCAGAAGAGAACTTATCTAGTGAGGA
TGAAGAGCTTCCCTGCTTCCAACACTTGTTATTTGGTAAAGTAAACAATATACCTT
CTCAGTCTACTAGGCATAGCACCGTTGCTACCGAGTGTCTGTCTAAGAACACAGA
GGAGAATTTATTATCATTGAAGAATAGCTTAAATGACTGCAGTAACCAGGTAATA
TTGGCAAAGGCATCTCAGGAACATCACCTTAGTGAGGAAACAAAATGTTCTGCTA
GCTTGTTTTCTTCACAGTGCAGTGAATTGGAAGACTTGACTGCAAATACAAACAC
CCAGGATCCTTTCTTGATTGGTTCTTCCAAACAAATGAGGCATCAGTCTGAAAGC
CAGGGAGTTGGTCTGAGTGACAAGGAATTGGTTTCAGATGATGAAGAAAGAGGA
ACGGGCTTGGAAGAAAATAATCAAGAAGAGCAAAGCATGGATTCAAACTTAGGT
GAAGCAGCATCTGGGTGTGAGAGTGAAACAAGCGTCTCTGAAGACTGCTCAGGG
CTATCCTCTCAGAGTGACATTTTAACCACTCAGCAGAGGGATACCATGCAACATA
ACCTGATAAAGCTCCAGCAGGAAATGGCTGAACTAGAAGCTGTGTTAGAACAGC
ATGGGAGCCAGCCTTCTAACAGCTACCCTTCCATCATAAGTGACTCTTCTGCCCTT
GAGGACCTGCGAAATCCAGAACAAAGCACATCAGAAAAAGCAGTATTAACTTCA
CAGAAAAGTAGTGAATACCCTATAAGCCAGAATCCAGAAGGCCTTTCTGCTGAC
AAGTTTGAGGTGTCTGCAGATAGTTCTACCAGTAAAAATAAAGAACCAGGAGTG
GAAAGGTCATCCCCTTCTAAATGCCCATCATTAGATGATAGGTGGTACATGCACA
GTTGCTCTGGGAGTCTTCAGAATAGAAACTACCCATCTCAAGAGGAGCTCATTAA
GGTTGTTGATGTGGAGGAGCAACAGCTGGAAGAGTCTGGGCCACACGATTTGAC
GGAAACATCTTACTTGCCAAGGCAAGATCTAGAGGGAACCCCTTACCTGGAATCT
GGAATCAGCCTCTTCTCTGATGACCCTGAATCTGATCCTTCTGAAGACAGAGCCC

CAGAGTCAGCTCGTGTTGGCAACATACCATCTTCAACCTCTGCATTGAAAGTTCC
CCAATTGAAAGTTGCAGAATCTGCCCAGAGTCCAGCTGCTGCTCATACTACTGAT
ACTGCTGGGTATAATGCAATGGAAGAAAGTGTGAGCAGGGAGAAGCCAGAATTG
ACAGCTTCAACAGAAAGGGTCAACAAAAGAATGTCCATGGTGGTGTCTGGCCTG
ACCCCAGAAGAATTTATGCTCGTGTACAAGTTTGCCAGAAAACACCACATCACTT
TAACTAATCTAATTACTGAAGAGACTACTCATGTTGTTATGAAAACAGATGCTGA
GTTTGTGTGTGAACGGACACTGAAATATTTTCTAGGAATTGCGGGAGGAAAATGG
GTAGTTAGCTATTTCTCATGATTTTGAAGTCAGAGGAGATGTGGTCAATGGAAGA
AACCACCAAGGTCCAAAGCGAGCAAGAGAATCCCAGGACAGAAAGATCTTCAGG
GGGCTAGAAATCTGTTGCTATGGGCCCTTCACCAACATGCCCACAGATCAACTGG
AATGGATGGTACAGCTGTGTGGTGCTTCTGTGGTGAAGGAGCTTTCATCATTCAC
CCTTGGCACAGGTGTCCACCCAATTGTGGTTGTGCAGCCAGATGCCTGGACAGAG
GACAATGGCTTCCATGCAATTGGGCAGATGTGTGAGGCACCTGTGGTGACCCGAG
AGTGGGTGTTGGACAGTGTAGCACTCTACCAGTGCCAGGAGCTGGACACCTACCT
GATACCCCAGATCCCCCACAGCCACTACTGA

BRCA1- amino acid sequence of exon18 skipping

MDLSALRVEEVQNVINAMQKILECPICLELIKEPVSTKCDHIFCKFCMLKLLNQKKGP
SQCPLCKNDITKRSLQESTRFSQLVEELLKIICAFQLDTGLEYANSYNFAKKENNSPEH
LKDEVSIIQSMGYRNRAKRLLQSEPENPSLQETSLSVQLSNLGTVRTLRTKQRIQPQKT
SVYIELGSDSSEDTVNKATYCSVGDQELLQITPQGTRDEISLDSAKKAACEFSETDVT
NTEHHQPSNNDLNTTEKRAAERHPEKYQGSSVSNLHVEPCGTNTHASSLQHENSSLL
LTKDRMNVEKAEFCNKSKQPGLARSQHNRWAGSKETCNDRRTPSTEKKVDLNADP
LCERKEWNKQKLPCSENPRDTEDVPWITLNSSIQKVNEWFSRSDELLGSDDSHDGES
ESNAKVADVLDVLNEVDEYSGSSEKIDLLASDPHEALICKSERVHSKSVESNIEDKIFG
KTYRKKASLPNLSHVTENLIIGAFVTEPQIIQERPLTNKLKRKRRPTSGLHPEDFIKKA
DLAVQKTPEMINQGTNQTEQNGQVMNITNSGHENKTKGDSIQNEKNPNPIESLEKES
AFKTKAEPISSSISNMELELNIHNSKAPKKNRLRRKSSTRHIHALELVVSRNLSPPNCTE
LQIDSCSSSEEIKKKKYNQMPVRHSRNLQLMEGKEPATGAKKSNKPNEQTSKRHDSD
TFPELKLTNAPGSFTKCSNTSELKEFVNPSLPREEKEEKLETVKVSNNAEDPKDLMLS
GERVLQTERSVESSSISLVPGTDYGTQESISLLEVSTLGKAKTEPNKCVSQCAAFENPK
GLIHGCSKDNRNDTEGFKYPLGHEVNHSRETSIEMEESELDAQYLQNTFKVSKRQSF
APFSNPGNAEEECATFSAHSGSLKKQSPKVTFECEQKEENQGKNESNIKPVQTVNITA
GFPVVGQKDKPVDNAKCSIKGGSRFCLSSQFRGNETGLITPNKHGLLQNPYRIPPLFPI
KSFVKTKCKKNLLEENFEEHSMSPEREMGNENIPSTVSTISRNNIRENVFKEASSSNIN
EVGSSTNEVGSSINEIGSSDENIQAELGRNRGPKLNAMLRLGVLQPEVYKQSLPGSNC
KHPEIKKQEYEEVVQTVNTDFSPYLISDNLEQPMGSSHASQVCSETPDDLLDDGEIKE
DTSFAENDIKESSAVFSKSVQKGELSRSPSPFTHTHLAQGYRRGAKKLESSEENLSSED

EELPCFQHLLFGKVNNIPSQSTRHSTVATECLSKNTEENLLSLKNSLNDCSNQVILAK
ASQEHHLSEETKCSASLFSSQCSELEDLTANTNTQDPFLIGSSKQMRHQSESQGVGLS
DKELVSDDEERGTGLEENNQEEQSMDSNLGEAASGCESETSVSEDCSGLSSQSDILTT
QQRDTMQHNLIKLQQEMAELEAVLEQHGSQPSNSYPSIISDSSALEDLRNPEQSTSEK
AVLTSQKSSEYPISQNPEGLSADKFEVSADSSTSKNKEPGVERSSPSKCPSLDDRWYM
HSCSGSLQNRNYPSQEELIKVVDVEEQQLEESGPHDLTETSYLPRQDLEGTPYLESGIS
LFSDDPESDPSEDRAPESARVGNIPSSTSALKVPQLKVAESAQSPAAAHTTDTAGYNA
MEESVSREKPELTASTERVNKRMSMVVSGLTPEEFMLVYKFARKHHITLTNLITEETT
HVVMKTDAEFVCERTLKYFLGIAGGKWVVSYFS*
