# Supplementary material for: Case Report: Functional validation of a rare variant BRCA1 c.5193 + 2dupT in a family with cancer history
Source: Front Oncol. 2025 Sep 30;15:1623700. doi: 10.3389/fonc.2025.1623700 (PMC12518100; doi:10.3389/fonc.2025.1623700)
Supplement: Supplementary file 1 [file DataSheet1.zip › Supplementary Material/Supplementary Material.pdf]

Supplementary Materials

Supplemental Figure caption:

Supplemental Figure 1: Genetic map of pcMINI-C-wt/mut minigene plasmid construction. The fragment including Exon 17 (78 bp) - Intron 17 (500 bp) - Exon 18 (41 bp) - partial Intron 18 (758 bp) was cloned into the pcMINI-C vector.

The construct details was as follows: Using human genomic DNA (gDNA) as the template, the 1414 bp wild-type fragment of pcMINI-C was amplified with the primers pcMINI-C-BRCA1-KpnI-F and pcMINI-CBRCA1-XhoI-R. The 784 bp 3' end fragment was amplified using the wild-type fragment of pcMINI-C as the template and the primers BRCA1-mut-F and pcMINI-C-BRCA1-XhoI-R. The 652 bp 5' end fragment was amplified using the wild-type fragment of pcMINI-C serving as the template and the primers pcMINI-C-BRCA1-KpnI-F and BRCA1-mut-R. The mutated fragment of pcMINI-C, which is 1415 bp in length, was amplified using a 1:1 mixture of the two end fragments as the template and the primers pcMINI-C-BRCA1-KpnI-F and pcMINI-C-BRCA1-XhoI-R. The specific primer sequences required for plasmid construction are provided in Supplementary Table 1.

Supplemental Tables

Supplemental Table 1. Primer sequences

Primer names	Primer Sequence (5’-3’)
pcMINI-C-BRCA1-KpnI-F	ACGATGACAAGCTTGGTACCATGCTGAGTTTG TGTGTGAA
pcMINI-C-BRCA1-XhoI-R	GCAGGGCAGTTTTCCTCGAGGGATATCCTGGT TTGCGCTG
BRCA1-mut-F	TGAATGAGGTTAAGTACTTGA
BRCA1-mut-R	TCAAGTACTTAACCTCATTCA
