# Supplementary figures and images for: Case Report: Functional validation of a rare variant BRCA1 c.5193 + 2dupT in a family with cancer history
Source: Front Oncol. 2025 Sep 30;15:1623700. doi: 10.3389/fonc.2025.1623700 (PMC12518100; doi:10.3389/fonc.2025.1623700)

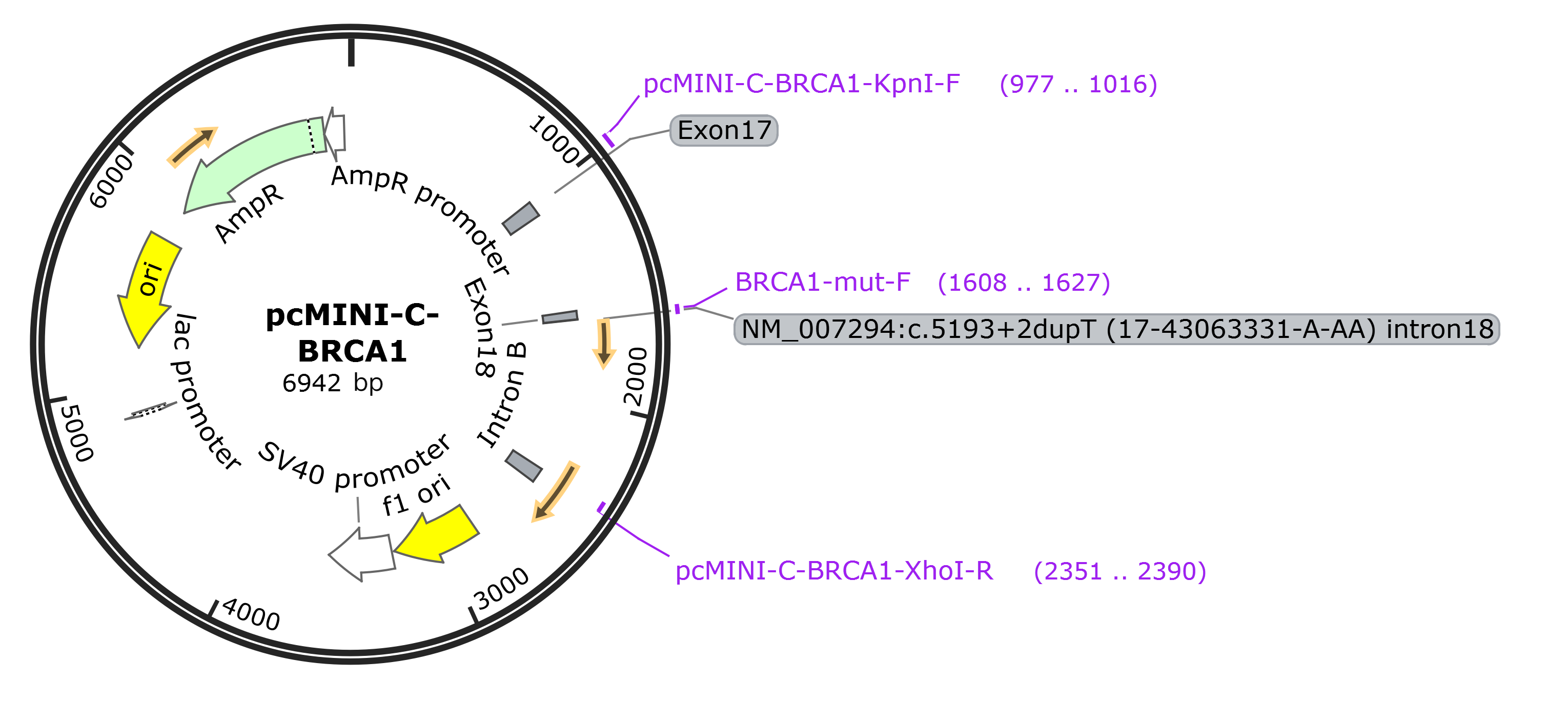

Supplement: Supplementary file 1 [file DataSheet1.zip › Supplementary Material/supplementary figure 1.tif]
